# Atomic-level insights into the high intrinsic thermostability of individual anatase TiO_2_ nanocrystals through surface-locking effects

**DOI:** 10.1038/s41467-026-73332-5

**Published:** 2026-05-20

**Authors:** Xiaoyun Guo, Yujing Zhang, Chao Yang, Yunhao Lu, Zimo Lin, Min Tang, Guanxing Li, Yang Ou, Beien Zhu, Ying Jiang, Zhong-kang Han, Wentao Yuan, Yi Gao, Ze Zhang, Yong Wang

**Affiliations:** 1 Center of Electron Microscopy and State Key Laboratory of Silicon and Advanced Semiconductor Materials, School of Materials Science and Engineering, Zhejiang University, Hangzhou, 310027 China; 2 Institute of Fundamental and Transdisciplinary Research, Zhejiang University, Hangzhou, 310027 China; 3 Zhejiang Key Laboratory of Low-Carbon Synthesis of Value-Added Chemicals, Zhejiang University, Hangzhou, 310027 China; 4 Photon Science Research Center for Carbon Dioxide, Shanghai Advanced Research Institute, Chinese Academy of Sciences, Shanghai, 201210 China; 5 Key Laboratory of Interfacial Physics and Technology, Shanghai Institute of Applied Physics, Chinese Academy of Sciences, Shanghai, 201800 China; 6 Zhejiang Province Key Laboratory of Quantum Technology and Device, School of Physics, Zhejiang University, Hangzhou, 310027 China

**Keywords:** Nanoparticles, Nanoparticles

## Abstract

Nanocrystal phase thermostability is critical for their applications, yet fundamentally governed by complex thermodynamic and kinetic variables. Understanding the stabilizing mechanisms and dominant factors requires atomic-level insights into dynamic evolution across surface and bulk regions under extreme conditions. Herein, we present a comprehensive in-situ investigation of individual single-crystalline anatase TiO_2_ nanorods using spherical aberration-corrected scanning transmission electron microscopy. By simultaneously acquiring environmental secondary electron images for surface topography and high-angle annular dark-field images for bulk atomic structures, we reveal the extraordinary phase stability of individual anatase nanorods governed by surface effects, distinct from aggregated nanorods. Anatase TiO_2_ nanorods undergo morphology reshaping and surface atomic reconstruction above 600 °C, involving transformation from high-index surfaces to (101) facets and the formation of (1 × 4)-reconstructed (001) surfaces. Remarkably, individual anatase TiO_2_ nanorods maintain the anatase structure even up to 1250 °C without transforming into the rutile phase. The restructuring lowers the total energy of the system, and acts as a kinetic “surface-locking” effect preventing rutile nucleation. Beyond elucidating the restructuring mechanisms and intrinsic thermostability of TiO_2_ nanocrystals, this work also establishes an effective pathway for simultaneously probing the complex structural evolution of nanomaterials across both surface and bulk regions.

## Abstract (web summary)

Nanocrystal phase thermostability is critical for several applications, yet stabilizing mechanisms under extreme conditions are not fully understood. Here, the authors give atomic insights into the high intrinsic phase stability of individual anatase TiO_2_ nanorods.

## Introduction

Precisely controlling the physical and chemical properties of materials through phase engineering has long been sought. These properties are fundamentally governed by crystalline phase structures, which are defined by specific atomic arrangements, bonds, symmetry, and thermodynamic parameters. Phase stability, particularly thermostability, which refers to the material’s ability to maintain its physical and chemical properties under thermal stress, is important for materials and devices, greatly affecting long-term reliability and efficiency in practical applications^1–9^. When external conditions change, phase transitions occur caused by fluctuations in the internal energy of nanomaterials, which is a common phenomenon that widely exists in nature and is used by various technologies, such as water vaporization in the water cycle, structural transition of shape memory alloys, information storage, superconducting transition of superconductors^6,10–14^. Fundamentally, phase thermostability is governed by a complex interplay of thermodynamic and kinetic variables (such as nucleation dynamics, atomic diffusion, and defect concentrations)^15–20^. Consequently, extensive research has been dedicated to elucidating the structural evolution under operating conditions at the atomic level and identifying the primary factors governing phase stability, ultimately aiming to design materials with high intrinsic robustness^21–24^.

Significant progress has been made in the study of phase stability and transition in bulk materials over the past few decades, and several strategies have been developed to enhance phase stability, including embedded particles, alloy particles, self-assembled nanocrystals and so on^25–30^. Compared to thermostability manipulation in bulk materials via interface engineering, much less is known about the role of surface effects in governing the phase stability of nanomaterials, where surfaces become increasingly influential as dimensions shrink^15–17^. However, current studies primarily focus on isolated surface atomic structure and properties themselves, leaving a substantial gap in understanding whether and how to actively utilize surface effects to enhance thermostability^31–35^. The inherent complexity of nanocrystal surface structures, which consist of facets, terraces, edges, and corners, makes it difficult to fully understand their interactions with the external environment^36,37^. Consequently, identifying the dominant factors among these complex variables requires in-situ experiments under realistic operating conditions to comprehensively compare the distinct structural evolutions and stabilities at different surface and bulk sites. Yet obtaining such site-specific, atomic-level structural information in real time at elevated temperatures remains an experimental challenge.

Recent advancements in environmental scanning transmission electron microscopy (ESTEM)^38–43^—particularly systems equipped with secondary electron (SE) detectors, provide an opportunity to explore surface effects on phase stability in real-time. By enabling the simultaneous acquisition of SE images for surface topography and high-resolution STEM images for bulk/interface atomic structures, this technique allows for a comprehensive in-situ investigation of structural evolution at both surfaces and interfaces^40–43^. As a classical model system widely studied over the past decades, the anatase-to-rutile transition in TiO_2_ poses a major challenge for achieving high thermal stability: the anatase phase, being inherently metastable and high-energy, favors transformation to rutile at high temperatures^44,45^. The transition to rutile occur at the elevated temperatures^25,29,32,46–48^, and anatase TiO_2_ with distinct thermostabilities in the separated experiments were attributed to various factors, such as impurities, sample preparation methods, defects, grain boundary, size, etc^25,29,32,45,47–50^., yet several critical issues remain elusive. In particular, the fundamental role of surfaces in determining the thermal stability of anatase nanocrystals remains unclear, making it challenging to accurately assess the intrinsic phase stability of individual TiO_2_ nanocrystals.

In the prior research, the surface atomic structure and properties of a variety of anatase TiO_2_ surfaces, such as (001) and (101), etc., have been systematically studied via both theoretical and microscopy methods^35,51–57^. Herein, to clarify the surface effect on the phase stability, we in situ explored the structural evolution of individual anatase TiO2 nanocrystals at the atomic level via spherical aberration-corrected (Cs-corrected) scanning transmission electron microscopy. The combined use of in-situ environmental secondary electron and high-angle annular dark-field (HAADF)-STEM imaging revealed both surface structures and dynamic mass-transport processes at the atomic scale. In-situ experiments revealed that individual anatase TiO_2_ nanorods exhibit high thermal stability, maintaining the anatase phase even at 1250 °C without transforming into rutile. The surface of anatase TiO_2_ undergoes morphology reshaping and a sequence of reconstructions with increasing temperature, ultimately leading to a stable (101) facet with lower surface energy. This low-energy surface plays a pivotal role in the phase thermal stability, which significantly stabilizes the metastable anatase phase via a “surface-locking” mechanism, which kinetically inhibits surface rutile nucleation.

## Results

### Surface evolution of individual anatase TiO_2_ nanorods

To comprehensively estimate the surface effect on the phase stability and correctly evaluate the intrinsic phase stability of individual TiO_2_ nanocrystals, we chose the anatase TiO_2_ nanorods as the model system, which are enclosed by several well-defined facets, including (100), (301), (101), and (001). The anatase TiO_2_ nanorods were synthesized by a two-step hydrothermal method^58^ (see Methods), which is exposed to several different surfaces and has well-defined crystallinity. Given the use of sodium-containing precursors during synthesis, several measures were taken to exclude the potential influence of Na contamination (see Supplementary Note 1). The synthesized nanorods were washed with deionized water to remove surface contaminants^59–61^. The presence of residual sodium was checked by several methods (Supplementary Figs. 1–4), including energy-dispersive X-ray spectroscopy (EDS), X-ray photoelectron spectroscopy (XPS), and electron energy-loss spectroscopy (EELS). No obvious Na signals were observed, indicating surface Na contaminants did not play a significant role in our study.

According to previous studies, there is a critical size in the anatase-to-rutile phase transition (14 to 45 nm), beyond which the rutile phase is generally thermodynamically more stable^44,49,50,62,63^. The micro-sized TiO_2_ particles investigated in this work far exceed the critical size. Consequently, based on size considerations alone, the system exhibits a thermodynamic preference for transforming into rutile. In the experiments, to evaluate the intrinsic phase stability of individual TiO_2_ nanocrystals, we first selected the individual nanocrystals that were not in contact with other nanocrystals to minimize the other structural factors involved in the experiments.

**Fig. 1:**
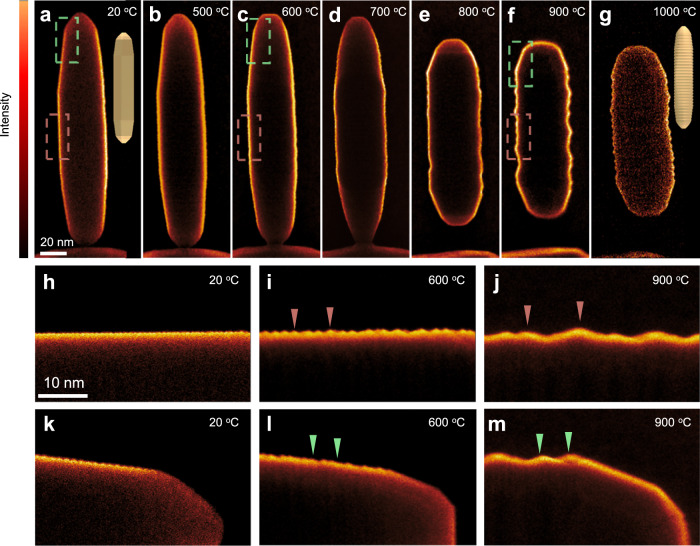
The morphology evolution of anatase TiO_2_ nanorod. In-situ HRSEM images of a typical anatase TiO_2_ nanorod in the heating process in vacuum (TEM column pressure: 5 × 10^-5 ^Pa). The heating rate of 1 °C s^‒1^, and each temperature should be maintained for a minimum duration of 30 min. **a–g** A typical morphology evolution process of the anatase TiO_2_ nanorod at different temperatures. **b–g** share the same scale bar as (**a**). **h–m** The enlarged HRSEM images of red and green box areas marked in (**a,** **c,** **f**) show the detailed morphology evolution at 20 °C (**a**), 600 °C (**c**), and 900 °C (**f**), respectively. Red and green arrows indicate protrusions on the surface of the TiO_2_ nanorods. **i–m** share the same scale bar as (**h**). The color scale is linear.

The in-situ heating experiments were performed to dynamically monitor the surface structural evolution of individual anatase TiO_2_ nanorods, from 20 to 1000 °C under vacuum (10^−5 ^Pa) at the heating rate of 1 °C s^−1^, and maintained at each temperature for at least 30 min. In-situ high-resolution scanning electron microscopy (HRSEM) images of the nanorods at different temperatures were acquired to show the morphology evolution of anatase TiO_2_ nanorods. From 20 to 500 °C, the morphology of the selected anatase TiO_2_ nanorod did not show a notable change (Fig. 1a, b). When the temperature exceeded 600 °C, the morphology of the nanorod also remained almost unchanged (Fig. 1c, d), with length and width unchanged compared to 20 °C ( ≈ 650 nm; ≈65 nm). While the surface of the nanorod started becoming no longer atomically flat, and some small protrusions appeared on the side surface of the nanorod, which indicated the possible surface reconstruction occurred. Above 700 °C, the morphology of the TiO_2_ nanorod exhibited significant change, the length became significantly shorter and the width slightly wider after heating at 1000 °C for more than 5 h (Fig. 1e–g). The small protrusions on the side surfaces gradually evolved into larger protrusions and the tip of the nanorod became flatter (as shown in the enlarged images in Fig. 1h–m). The observations of another two individual nanorods showed a similar process up to 1000 and 1200 °C, respectively (Supplementary Figs. 5, 6, Supplementary Movie 1).

### Atomic structural evolution of the anatase TiO_2_ nanocrystals

To observe the atomic structural evolution of the anatase TiO_2_ nanocrystals at elevated temperatures, we performed the in-situ heating experiments in a Cs-corrected scanning transmission electron microscope. The in-situ experiments were performed in vacuum and gas conditions via the Cs-corrected scanning transmission electron microscopes (Titan G2 80-200, Hitachi HF-5000), with micro-electromechanical system-based heating holders (DENS solutions DH30 double-tilt heating holder, Hitachi single-tilt heating holder). Since the TiO_2_ crystal is very sensitive to electron beam (e-beam) irradiation, several measurements have been adopted to minimize the e-beam effect in the experiments. During the in-situ experiment, a low dose (≈0.6 nA) was used, and the electron beam was turned on only during photo acquisition to reduce its influence on the sample^51,55^.

**Fig. 2:**
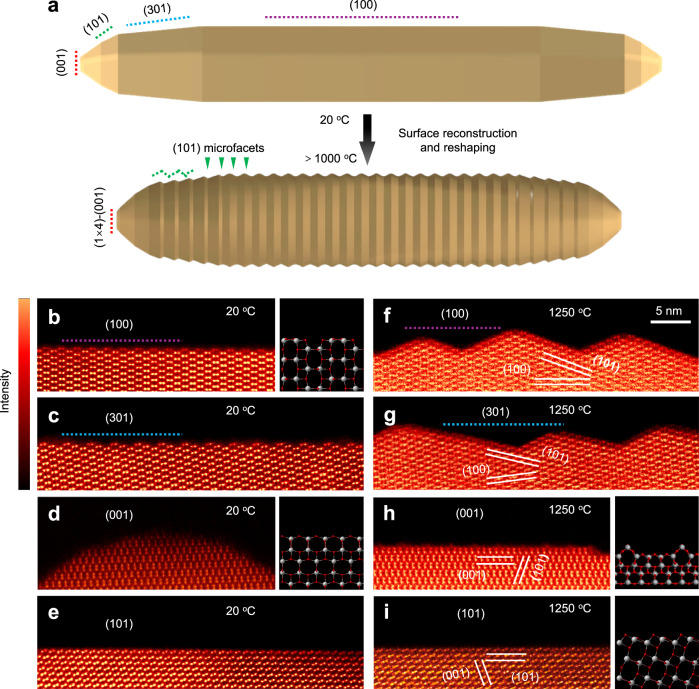
In-situ HAADF STEM images show the structural evolution of TiO_2_ nanorod at high temperatures in vacuum (column pressure: 10^‒5 ^Pa), viewed along the [010] direction. **a** Schematic diagram of morphology changes of a typical anatase TiO_2_ nanorod before and after heating. **b–e** The atomic-level HAADF STEM images of (100) (**b**), (301) (**c**), (001) (**d**), and (101) (**e**) surfaces at 20 °C. The insets in (**b, d**) are the corresponding atomic structural models of (100), and (001), respectively (Ti, gray; O, red). **f**–**i** The in-situ atomic-level HAADF STEM images show the surface reconstructions of (100) (**f**), (301) (**g**), (001) (**h**), and (101) (**i**) surfaces at 1250 °C. The insets in (**h, i**) are the atomic structural models of ADM model of (1 × 4) reconstructed (001) surface, and (101), respectively (Ti, gray; O, red). **c–i** share the same scale bar as (**b**). The color scale is linear. The dotted lines of different colors denote the respective crystallographic planes.

Figure 2a shows the schematic diagram of a typical as-synthesized anatase TiO_2_ nanorod. The size of the nanorod is about 650 nm long and 65 nm wide. The axial direction of the nanorod is along the [001] direction, and the sidewall is mainly exposed by (100) surfaces with atomic flatness (Fig. 2b). The tip of the nanorod is curved, which exposed by some high-index surfaces, such as (301) facet with an orderly stepped structure containing succession of a (101) terrace separated by (001) step, (101) facet which is exposed by both 6- and 5-fold coordinated Ti atoms (Ti_6c_ and Ti_5c_), with 2- and 3-fold oxygen atoms (O_2c_ and O_3c_) located on the ridge of the sawtooth-like structure, (001) facet exposed by 5-fold coordinated Ti (Ti_5c_) atoms, 3-fold coordinated O atoms (O_3c_), and 2-fold coordinated O (O_2c_) atoms, etc. (Fig. 2c–e), as well documented in refs. ^51–54^. The two-dimensional crystalline lattice 0.35 nm (101) and 0.37 nm (100) indicates the anatase phase. The prepared nanorods had good crystallinity, atomically flat surfaces, and few defects, providing a good platform for in situ study.

The phase transition points are range from 400 to 1200 °C, investigated by both ex-situ and in-situ routes^25^. In our experiments, it is found that anatase phase TiO_2_ nanorods could still maintain anatase phase even at a high temperature of 1250 °C after heating for more than 5 h. Compared to the as-synthesized anatase TiO_2_ nanorods, both the morphology and surface atomic structure show significant reconstruction (Fig. 2a). The originally flat surface of the selected nanorod became uneven, and the initial atomically flat (100), (301) surfaces are reconstructed into protrusions formed by (101) facets with (001) surface transformed into the (1 × 4) reconstruction of ADM model (Fig. 2f–i), which is consistent with the previous studies. While we carefully checked the HAADF STEM images in different areas, we did not find the nucleation of the rutile phase. Given that the nanorod is significantly larger than the critical size, the phase transition is expected to occur readily. Therefore, the in-situ observations indicate anatase TiO_2_ nanorod exhibits a high intrinsic phase stability, which have been repeatedly confirmed in several cases (Supplementary Figs. 5–7).

**Fig. 3:**
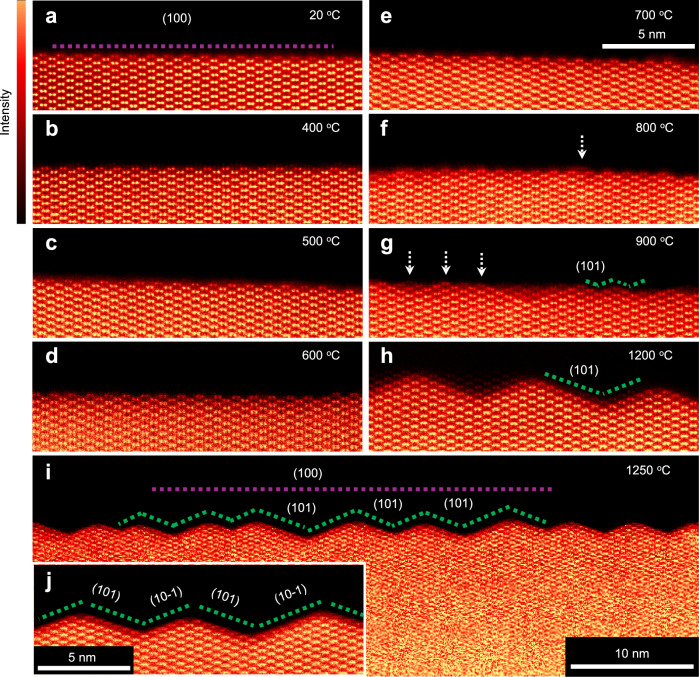
The structural evolution of anatase TiO_2_ nanorod (100) surface. **a–h** The in-situ HAADF STEM images show the atomic structural evolution of anatase TiO_2_ (100) surface at different temperatures, in vacuum (column pressure: 5 × 10^−5 ^Pa). **i** The atomic level HAADF STEM image acquired at 1250 °C. **b–h** share the same scale bar as (**a**). **j** The enlarged image of (**i**). Each temperature should be maintained for a minimum duration of 30 min. The color scale is linear.

Since the morphology and surface structure show significant restructuring during the heating experiments, the in-situ STEM images were acquired to illustrate the detailed atomic evolution process of surface structures. Since the nanorod's surface is mainly exposed through (100), (301), (001), and (101) facets, the atomic structural evolution on each surface is decoupled. Initially, the (100) surface showed a bulk-truncated surface structure (Fig. 3a). During the heating process, this atomically flat surface did not show visible change from room temperature to ≈ 600 °C (Fig. 3a–d). When the temperature was beyond 600 °C (Fig. 3e, f), some fresh fuzzy islands began to appear on the surface (marked by the arrow), indicating that some atomic migration occurred. At 900 °C (Fig. 3g), the originally atomically flat (100) surface became obviously rough, with some fresh atomic columns appearing on the surface (white arrows) and some surface atomic columns disappearing. With further temperature elevation, these small protrusions grew higher and became sharpened (Fig. 3h), with (101) surface dominated. Finally, the (100) surface was completely reconstructed into well-arranged sharp (101) faceted protrusions (mean height: 1.2 nm; interval distance along [001]: 6.4 nm), as the temperature reached 1250 °C (Fig. 3i, j).

**Fig. 4:**
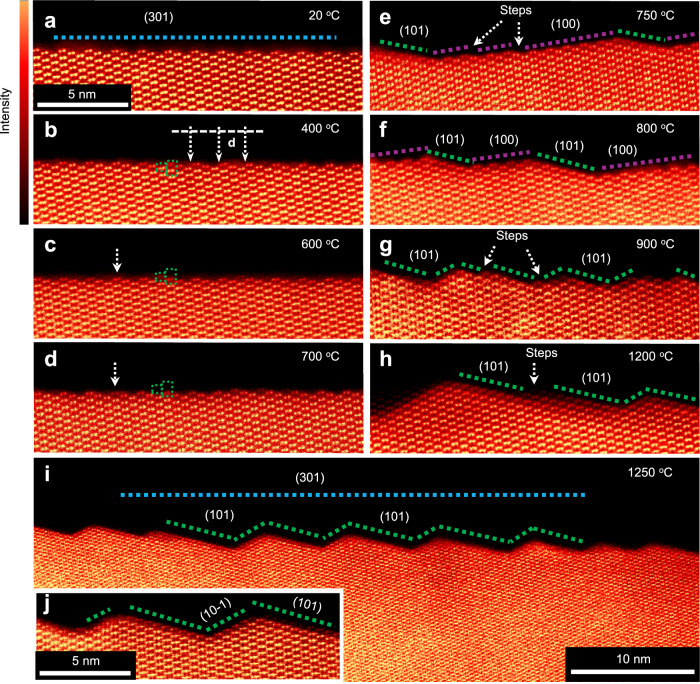
The structural evolution of anatase TiO_2_ nanorod (301) surface. **a–h** The in-situ HAADF STEM images show the atomic structural evolution of anatase TiO_2_ (301) surface at different temperatures, in vacuum (column pressure: 5 × 10^−5 ^Pa). **b–h** share the same scale bar as (**a**). **i** The atomic level HAADF STEM image acquired at 1250 °C. **j** The enlarged image of (**i**). Each temperature should be maintained for a minimum duration of 30 min. “d” indicating a typical periodicity of the 1 × 1 bulk-terminated (301) surface. The color scale is linear.

Compared with the reconstruction of the (100) surface, directly evolving into (101) surface protrusion, the structural evolution process of the (301) surface was more complex (Fig. 4a–j), which experienced two stages. Initially, the (301) surface showed an orderly stepped structure, which could be obtained by a succession of a (101) terrace with three titanium atom pairs, separated by a (001) step. In the first stage, this stepped (301) surface remained nearly unchanged from room temperature to 400 °C (Fig. 4a, b). At higher temperatures, the atoms on the surface of the nanorods gradually became unsharp and blurry. As indicated by the white arrow in Fig. 4c, some atomic columns became blurred or even disappeared at 600 °C, indicating that surface atomic migration occurred, as shown by the atoms circled in the green dotted box. The surface reconstruction became more obvious at 700 °C (Fig. 4d). As a result, at higher temperatures (750 and 800 °C), the (301) surface gradually transformed from a stepped (101) terrace (Fig. 4b) into a different stepped structure (Fig. 4e, f) consisting of (100) terraces and (101) facets, in the first stage.

In the following heating process (the second stage), the newly formed (100) terraces also became unstable and tended to reconstruct into (101) surface-dominated protrusions as depicted in Fig. 3. Therefore, at higher temperatures, with the formation of (101) faceted protrusions, the proportion of (100) surface significantly reduced (Fig. 4g), and eventually disappeared (Fig. 4h). The protrusions also grew larger and became more sharpened, eventually (301) surface was also completely transformed into a stepped structure dominated by the (101) surfaces at 1250 °C (Fig. 4i, j). Compared to the reconstructed (100) surface, the (101) protrusions formed by reconstructing the (301) surface exhibit a preferential orientation: (101) >  (10-1).

In addition, the (001) surfaces of the anatase TiO_2_ nanorod tip were kept bulk-truncated structure from room temperature to 600 °C, and then transformed into the (1 × 4) reconstruction by an ADM model, which could be stabilized at even 1250 °C (Supplementary Fig. 8), as discussed in ref. ^24^. At last, the structural evolution of (101) surface was also explored. While different from the significant reconstructions of other surfaces, the (101) remain stable and did not show significant reconstruction within 1250 °C, which confirms that the surface of (101) is the thermodynamically stable surface of anatase TiO_2_ (Supplementary Fig. 9). Overall, the morphology and the surface structures were reconstructed: (100) and (301) were reconstructed into stepped (101) surface; (001) was reconstructed via the ADM model.

### Real-time mass-transport dynamics of anatase TiO_2_ nanorods

**Fig. 5:**
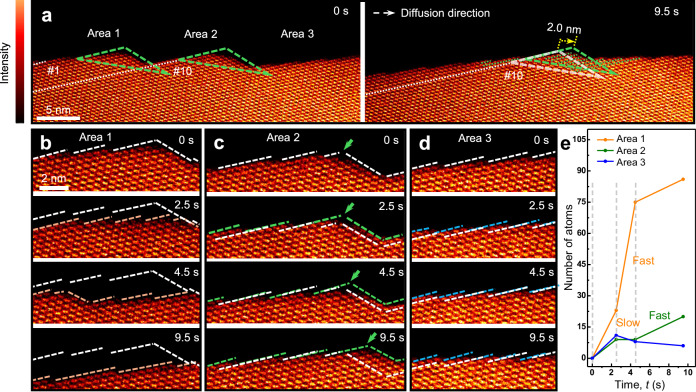
Selected snapshots from a STEM movie tracking the mass transportation during the dynamic process of surface diffusion of a typical anatase TiO_2_ nanorod (Supplementary Movies 2 and 3). The in-situ STEM images were collected at 1000 °C in vacuum (column pressure: 5×10^‒5 ^Pa), viewed from [010] direction. **a** Sequential HAADF STEM images of the anatase TiO_2_ surface, acquired at 0, and 9.5 s, collected from Supplementary Movie 2. The green dashed triangle frames indicate the change in the position of the step tip. The white dashed lines denote atoms in the nth row, whereas the green dashed lines indicate the newly incorporated atoms. The yellow dotted lines show the moving direction and distance of the step enclosed by the green dashed triangle frame. **b–d** The enlarged sequential HAADF STEM images of the anatase TiO_2_ surface, acquired at 0, 2.5, 4.5, and 9.5 s, collected from Supplementary Movie 2, corresponding to areas 1 (**b**), 2 (**c**), and 3 (**d**) of (**a**). **c, d** share the same scale bar as (**b**). The dotted white lines and color lines indicate the positions of atomic arrangement on the surface at initial and after diffusion respectively. The green arrows indicate the change in the position of the step tip. The color scale is linear. **e** Quantification of the changes in the number of atoms in areas 1, 2, and 3 of (**a**). Source data are provided as a Source Data file.

To further reveal why and how these reconstructions happen during the reshaping of anatase TiO_2_ nanorod, time-resolved STEM imaging was employed to track real-time mass-transport dynamics. Initially, two distinct steps existed on the surface, as framed in dotted green lines in Fig. 5a. Over time, the left step diminished, and even nearly vanishing at *t* = 9.5 s shown in Fig. 5a. Whereas, the right step sharpened through atomic accumulation and migrated ≈2.0 nm rightward (green dotted lines denote diffusion-mediated atomic additions). The diffusion process features synchronized atom depletion in area 1 and the accumulation of atoms in areas 2 and 3, as visualized in Fig. 5b–d (white and colored dotted lines mark pre- and post-diffusion atomic configurations, respectively). The detailed information was shown in the kinetics curve of the atomic diffusion in Fig. 5e. On the one hand, the reduction of atoms in area 1 was the fastest and nonlinear, where the rapid migration of atoms along the atomic layer in the period of *t* = 2.5 - 4.5 s led to the rapid reduction of atoms layer by layer in area 1 shown in Fig. 5b. On the other hand, the accumulation of atoms in areas 2 and 3 were different. In area 2, the increase of atoms was slow at first. After *t* = 4.5 s, atoms began to accumulate rapidly, indicating that nucleation was more difficult, but once nucleated, the step grew faster and gradually sharpened as shown in Fig. 5c. At *t* = 9.5 s, the atoms rapidly accumulated 3 layers along the original atomic layer, leading to the step sharper, with the lattice plane on both sides composed of (101) facets. In contrast, there was no significant accumulation of atoms in area 3 shown in Fig. 5d, just the length of the layers added along the existing atomic layer ladder changed repeatedly over time. In addition, the number of atoms decreased in area 1 was much greater than the number of atoms that increased in area 2, indicating that the excess atoms migrated layer by layer rapidly to the right along the atomic steps of area 3.

The mass transportation direction is consistent with the structural evolution showed in Supplementary Movie 3, where atoms migrate from the nanorod tip toward its midsection. The diffusion process ultimately leads to atom accumulation at steps, forming sharp protrusions with low-surface-energy (101) facets, resulting in the morphological evolution depicted in Fig. 1. The synergistic changes in (101) faceted step sites also indicate the mass transport occurs via surface diffusion, and the simultaneous growth and dissolution of the (101) facets have less energetic barriers than that of rutile-phase nucleation. Throughout the process, the rapid diffusion of surface atoms also suggests that, under our experimental conditions, atoms could migrate freely and stabilize directionally at low-energy sites. Therefore, it can be concluded that the absence of phase transformation under high-temperature conditions is not due to a lack of source materials or limitations in mass transport, but rather is kinetically constrained by the failure to form rutile nuclei on the low-energy anatase TiO_2_ surfaces.

## Discussion

A critical question arises regarding the origin of the high phase stability of the individual anatase TiO_2_ single-crystal nanorods, which can be sustained at exceptionally high temperatures up to 1250 °C. Notably, considering that the critical size between the anatase phase and rutile phase is in a range of 14-45 nm^25,34,49,50,62,63^, the size of our sample exceeds this range, it is anticipated the transition should be more favorable for the anatase to rutile phase transition. On the other hand, considering our experiments were performed in vacuum conditions, which are also considered to promote phase transformation by facilitating oxygen vacancy formation^64,65^. Even under prolonged extreme conditions (e.g., temperature: 1100 °C, duration å 10 h), the individual single-crystalline anatase nanorod retain the anatase phase (Supplementary Figs. 10–14). Thus, the exhibited high phase stability of the individual anatase single-crystal nanorod represents an anomalous phenomenon significantly different from the conventional understanding.

The phase stability of anatase has been studied extensively over the past 30 years, both theoretically and experimentally^25,32,34,66–72^, and multiple nucleation pathways have been proposed. For instance, Zhang and Banfield et al. revealed that rutile nucleates at interfaces and/or free surfaces of anatase particles with different particle packing; Lu et al. observed that the anatase-to-rutile transition of microcrystals is dominated by surface nucleation and growth^32,34^. Among these mechanisms, nucleation occurring via a (112) twin boundary in anatase is widely accepted^73–75^. However, the exact nucleation mechanisms and dominant factors governing phase stability remain controversial^34,46,50,63,73^, due to a lack of atomic-level dynamic evidence. In-situ observations reveal significant surface reconstruction via rapid atomic diffusion under our experimental conditions (Fig. 5; Supplementary Movies 2 and 3), confirming that mass transport is not a limiting factor. This observation actively rules out the previously proposed “structure-stuffing” effect^29^, which posits that titanium interstitials retard the phase transformation by impeding atomic diffusion.

**Fig. 6:**
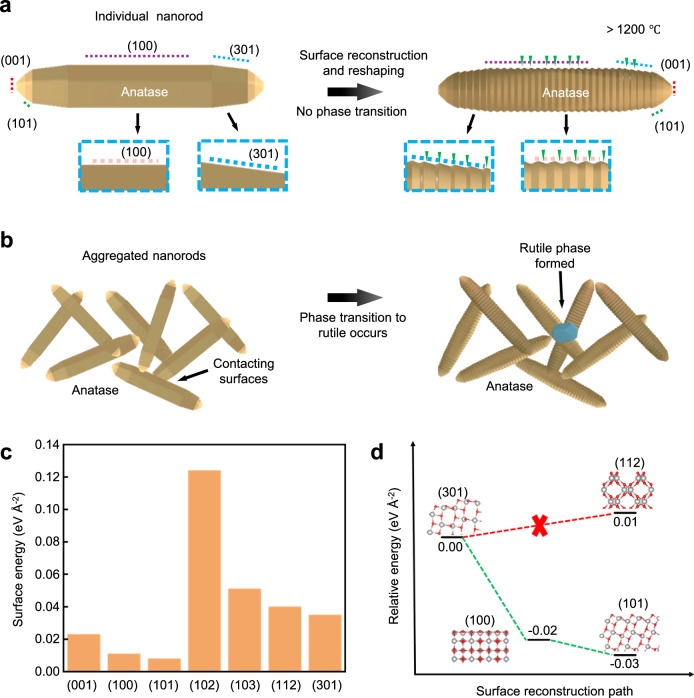
Schematic diagram illustrating the surface reconstruction pathway. **a, b** Schematic diagram of morphology changes of single anatase TiO_2_ nanorod (**a**), and multiple nanorods (**b**) before and after heating. **c** Surface energies of different surface cuts. **d** Schematic diagram of the surface reconstruction path.

Thermodynamically, the (100) and (301) surfaces on the individual TiO_2_ nanorod preferentially undergo reconstruction into the (101) surface. Surface energy calculations reveal that the (101) surface has the lowest energy (0.008 eV Å^‒2^), as shown in Fig. 6c, making it the most stable surface. DFT + *U* calculations further confirm that ${E}_{{{{\rm{surface}}}}}$(301) > ${E}_{{{{\rm{surface}}}}}$ (100) > ${E}_{{{{\rm{surface}}}}}$ (101), indicating a favorable pathway for this reconstruction (Fig. 6d). The (101) surface with very low surface energy, significantly prevents the surface rutile nucleation for the elevated barrier. Additionally, the rapid diffusion of surface atoms avoids the accumulation of atoms on the surface, which further reduces nucleation probability.

Furthermore, in our additional control experiments, a completely different phenomenon was found, where the phase transition occurred within several minutes when many TiO2 nanorods were stacked together. The nucleation of rutile crystallite formed at the multi-grain boundaries of adjacent particles, as shown in Supplementary Fig. 15. Within a certain range, only a single rutile crystal nucleates and grows, and polycrystalline rutile formation is not observed, which also implies that kinetic nucleation is an exceedingly difficult process, requiring the stacking of anatase TiO_2_ nanorods in a specific manner. Therefore, we conclude that the absence of phase transformation under high-temperature conditions is not limited by a lack of source materials or mass transport but rather is kinetically constrained by the failure to form rutile nuclei on the low-energy anatase TiO_2_ surfaces.

The distinct phase-transition behaviors observed between isolated and aggregated nanorods indicate that rutile nucleation could occur at the interface, and the surface reconstruction could play a critical role in determining its stability. As illustrated in Supplementary Fig. 16, the (112) twin boundary could be formed by specific edge-to-edge connections between two (101) facets. In individual nanorod experiments, the formation kinetics of this nucleation site is extremely challenging. After the reconstruction, the individual anatase TiO_2_ nanocrystal is mainly exposed by the (101) surfaces, significantly prevents the surface rutile nucleation or (112) surface formation (surface energy of 0.04 eV Å^‒2^) for the elevated barrier (Fig. 6). Conversely, the preferential rutile nucleation site, (112) twin boundary could be only formed when multiple nanorods stack in the specific configuration (Supplementary Fig. 16). As the number of stacked nanorods increases, the probability of (112) twin boundary formation increases, and thus the probability of phase transformation also increases. By in-situ investigating a well-designed model system in our experiments—single-crystal nanorods with uniform dimensions, well-defined morphology, and high crystallinity—we effectively decoupled the influence of surface structure and restructuring in its phase stability from other contributing factors.

Our discovery provides atomic-scale insights into the critical influence of surface atomic structure and restructuring on the phase stability of nanomaterials. Additionally, the rapid diffusion of surface atoms avoids the accumulation of atoms on the surface, which further reduces nucleation probability. These combined effects significantly increase the nucleation barrier during the phase transition, resulting in the abnormal thermal stability of the anatase phase. We refer to this phenomenon as surface locking. This finding highlights the critical impact of surface on the stability of nanomaterials, which significantly affects the secondary phase nucleation and mass transport in the phase transformation of nanomaterials. The high thermal stability of anatase TiO_2_ nanocrystals at 1250 °C achieved through “surface locking” reveals the distinct surface dynamics of nanoparticles and their profound impact on phase stability and transformation kinetics.

In summary, through an in-situ atomic-scale exploration of both surface and bulk structural evolution of anatase TiO_2_ nanocrystals, we revealed the intrinsic phase stability of the individual anatase TiO_2_ single-crystal nanorod, which could maintain the anatase phase structure at 1250 °C. In the heating process, different from the expected anatase-to-rutile phase transition, only the surface reconstructions and reshaping of the individual anatase TiO2 nanorod occurred, gradually reconstructing into the stable structures mainly exposed by (101) surfaces with decreased surface energy. The restructuring not only reduces the total energy of the system, but also kinetically inhibits the surface rutile nucleation, leading to the abnormal phase stability of the anatase TiO2 at a 1250 °C high temperature. Our results reveal atomic-scale insights into the critical impact of surface atomic structure and restructuring on the phase stability of nanomaterials, and reveal the high intrinsic phase stability of anatase TiO_2_ could be achieved via a “surface-locking” effect. Furthermore, this work establishes a framework for simultaneously probing the complex structural dynamics across both surface and bulk regions for nanomaterials.

## Methods

### Materials

Sodium hydroxide (NaOH, >98%) was purchased from Shanghai Aladdin Biochemical Technology Co., Ltd. TiO_2_ (P25, 25 nm) was purchased from Sinopharm Chemical Reagent Beijing Co., Ltd.

### Synthesis of anatase TiO_2_ nanorods

The anatase TiO_2_ nanorods were synthesized via a hydrothermal method reported in ref.^58^. In a typical synthesis, 1 g of TiO_2_ (P25) was dissolved in 40 mL of 10 M NaOH solution. The mixture was transferred into a 50 mL Teflon-lined stainless-steel autoclave, maintained at 120 °C for 24 h. After naturally cooling to the room temperature, the precipitates (Na-titanate) were collected by centrifugation at 3000 × *g* and washed with deionized water several times. Then, 1 g Na-titanate (wet, isolated by centrifugation without drying) were dispersed into 40 mL deionized water solution, then transferred to a 50 mL Teflon-lined stainless-steel autoclave, and sealed. The autoclave was put into an oven, heated at 200 °C for 24 h, and cooled naturally in air, producing white tetragonal-faceted nanorods precipitates. These white precipitates were isolated from the solution by centrifugation at 3000× *g* and subsequently washed with deionized water several times, and finally dried at 60 °C in vacuum for 10 h. The X-ray photoelectron spectroscopy (XPS) spectra was collected on Kratos Axis Supra + . The anatase TiO_2_ nanorods models were created using Computer-Aided Design software (CAD) by SolidWorks to facilitate an intuitive display of the samples.

### In-situ STEM observations

The in-situ STEM experiments were conducted in an Titan G^2^ 80-200 scanning transmission electron microscope (Thermo Fisher, 200 kV), with a Cs-corrected and a high spatial resolution (≈0.8 Å). Before the test, to ensure high-quality imaging, all low-order aberrations (up to order 5) have been calibrated to an acceptable level, such as Cs <0.5 μm, A1 (2-fold astigmatism) <2 nm, A2 (3-fold astigmatism) <20 nm, and B2 (coma) <20 nm. The convergence angle employed for STEM imaging was ≈21 mrad. The annular detection angle for HAADF image is set to ≈53-200 mrad, while for bright-field (BF) image, it is adjusted to ≈0-20 mrad.

During the in-situ STEM experiments, the as-obtained TiO_2_ nanorods are dispersed in ethanol and dropped on the heating chips, which are then transferred into the TEM chamber via a Wildfire D6 double tilt heating holder (DENS solutions). Instead of directly heating our sample to the elevated temperatures, in the step-by-step heating process, the temperature interval was set to 100 °C with a heating rate of ≈5 °C s^‒1^.

TiO_2_ is usually very sensitive to e-beam irradiation and it is easy to undergo irradiation decomposition under irradiation^76,77^. In the STEM mode, the radiation damage of the e-beam will be more severe because the electron beam is convergent and has a higher beam current density. To minimize the radiation damage of the electron beam to the maximum extent, we take a series of measures to reduce the radiation dose of the electron beam in the sample (≈0.6 nA). In particular, the target surface area was moved under the electron beam only during the imaging process.

### In-situ HRSEM observations

HRSEM analysis of TiO_2_ nanorods was performed in a field emission environmental scanning transmission electron microscope (HF5000, Hitachi company), operating at 200 kV, with gas environment available. The TiO_2_ nanorods were dispersed on a heating chip, and then loaded into the ESTEM through a single-tilt heating holder (a MEMS holder) with a heating rate of ≈1 °C s^‒1^.

### In-situ ETEM observations

ETEM experiment was performed in an environmental transmission electron microscope (Hitachi H-9500) operated at 300 kV equipped with a Gatan One view Camera and an external gas delivery system. A double tilt heating holder (Chipnova, CNT-SHBO-D) is used in our experiments. The samples were then heated from 20 °C to the target temperatures with a heating rate of 5 °C·s^‒1^. At each scheduled temperature point, the samples were kept on at least 10 min for detailed observations. In addition, in order to minimize the radiation damage of the e-beam in the experiment, the gun valve was only opened when taking pictures.

### Statistics and reproducibility

The sample size was determined based on established standards in the field of materials science and TEM. In this study, all in-situ heating experiments were independently repeated at least 10 times with consistent results, and more than 100 individual anatase nanorods were examined.

### DFT calculations

The spin-polarized DFT calculations were performed using the Perdew-Burke-Ernzerh (PBE) functional to optimize all surface structures, implemented in the Vienna ab initio simulation package (VASP). Projector-augmented wave (PAW) potentials were used to represent core-valence interactions. To account for the localized Ti *d* states, we applied DFT + *U* with an effective *U* value of 4.1 eV. During optimization, atomic positions were relaxed until residual forces were below 0.02 eV Å^‒1^, and the convergence criterion for electronic self-consistent energy was set to 10^‒4 ^eV. A plane-wave cutoff of 400 eV was used to expand the Kohn-Sham valence states, and a vacuum layer of 10 Å was included to prevent interactions between periodic images. Surface energies of perfect anatase (001), (100), (101), (102), (103), (112), and (301) surfaces were calculated using (1 × 1) slab models with corresponding K-point grids up to (10 × 10×1), (10 × 4 × 1), (6 × 10 × 1), (3 × 10 × 1), (5 × 10 × 1), (8 × 4 × 1), and (2 × 9 × 1), respectively.

The surface energy $({E}_{{{{\rm{surface}}}}})$ is given by:

1 $${E}_{{{{\rm{surface}}}}}=\frac{{E}_{{{{\rm{slab}}}}}-{{n\; E}}_{{{{\rm{bulk}}}}}}{2{A}_{{{{\rm{slab}}}}}}$$

where ${E}_{{{{\rm{slab}}}}}$ is the total energy of the slab, ${E}_{{{{\rm{bulk}}}}}$ is the energy per atom in the bulk structure, *n* is the number of atoms in the slab, and ${A}_{{{{\rm{slab}}}}}$ is the surface area of the slab. The factor of 2 accounts for the two surfaces present in the slab model.

## Supplementary information

Suppplementary Information

Description of Additional Supplementary Files

Supplementary Movie 1

Supplementary Movie 2

Supplementary Movie 3

Transparent Peer Review file

## Source data

Source Data

## Data Availability

The data that support the findings of this study are available from the corresponding authors upon request. Source data are provided with this paper.
