# Supplementary material for: Atomic-level insights into the high intrinsic thermostability of individual anatase TiO2 nanocrystals through surface-locking effects
Source: Nat Commun. 2026 May 20;17:6658. doi: 10.1038/s41467-026-73332-5 (PMC13381851; doi:10.1038/s41467-026-73332-5)
Supplement: Supplementary file 1 — Suppplementary Information [file 41467_2026_73332_MOESM1_ESM.pdf]

Supplementary Information

Atomic-level insights into the high intrinsic thermostability of individual anatase TiO_2 nanocrystals through surface-locking effects

Xiaoyun Guo[1†], Yujing Zhang[2,3†], Chao Yang[4], Yunhao Lu[4,5], Zimo Lin[1], Min Tang[1], Guanxing Li[1], Yang Ou[1], Beien Zhu[2,3], Ying Jiang[1], Zhong-kang Han[1], Wentao Yuan[1*], Yi Gao[2,3], Ze Zhang[1] and Yong Wang[1*]*

[1]Center of Electron Microscopy; State Key Laboratory of Silicon and Advanced Semiconductor Materials; School of Materials Science and Engineering; Institute of Fundamental and Transdisciplinary Research; Zhejiang Key Laboratory of Low-Carbon Synthesis of Value-Added Chemicals, Zhejiang University, Hangzhou, 310027, China

[2]Photon Science Research Center for Carbon Dioxide, Shanghai Advanced Research Institute, Chinese Academy of Sciences, Shanghai, 201210, China

[3]Key Laboratory of Interfacial Physics and Technology, Shanghai Institute of Applied Physics, Chinese Academy of Sciences, Shanghai, 201800, China

[4]State Key Laboratory of Silicon and Advanced Semiconductor Materials, School of Materials Science and Engineering, Zhejiang University, Hangzhou, 310027, China

[5]Zhejiang Province Key Laboratory of Quantum Technology and Device, School of Physics, Zhejiang University, Hangzhou, 310027, China

[†]These authors contributed equally to this work.

[*]Corresponding author. Email: wentao_yuan@zju.edu.cn; hanzk@zju.edu.cn; yongwang@zju.edu.cn;

Table of Contents

1. Supplementary Figures (1-16)

Supplementary Note 1. Potential influence of Na contamination

In EDS, XPS and EELS data, no obvious Na signals were observed (**Supplementary Figs. 1-4**), indicating that Na contamination did not play a significant role in our study. In the EDS analysis, no distinct signal corresponding to the Na element was observed within its characteristic energy range (1.04–1.06 keV), as the signal level remained consistent with the background (**Supplementary Fig. 1**). In the XPS spectrum, the peak position attributed to Na (1072 eV) is in close proximity to the Ti Auger electron peak (1067 eV); based on binding energy comparison, this feature was assigned to the Auger peak of Ti rather than Na (**Supplementary Fig. 2**). To further investigate the presence of Na, EELS measurements were conducted at multiple locations across different nanorods (**Supplementary Figs. 3-4**). No discernible signal was detected at the expected energy position for Na (1072 eV). By integrating results from EDS, XPS, and EELS analyses, the potential influence of Na contamination was systematically evaluated and ruled out.

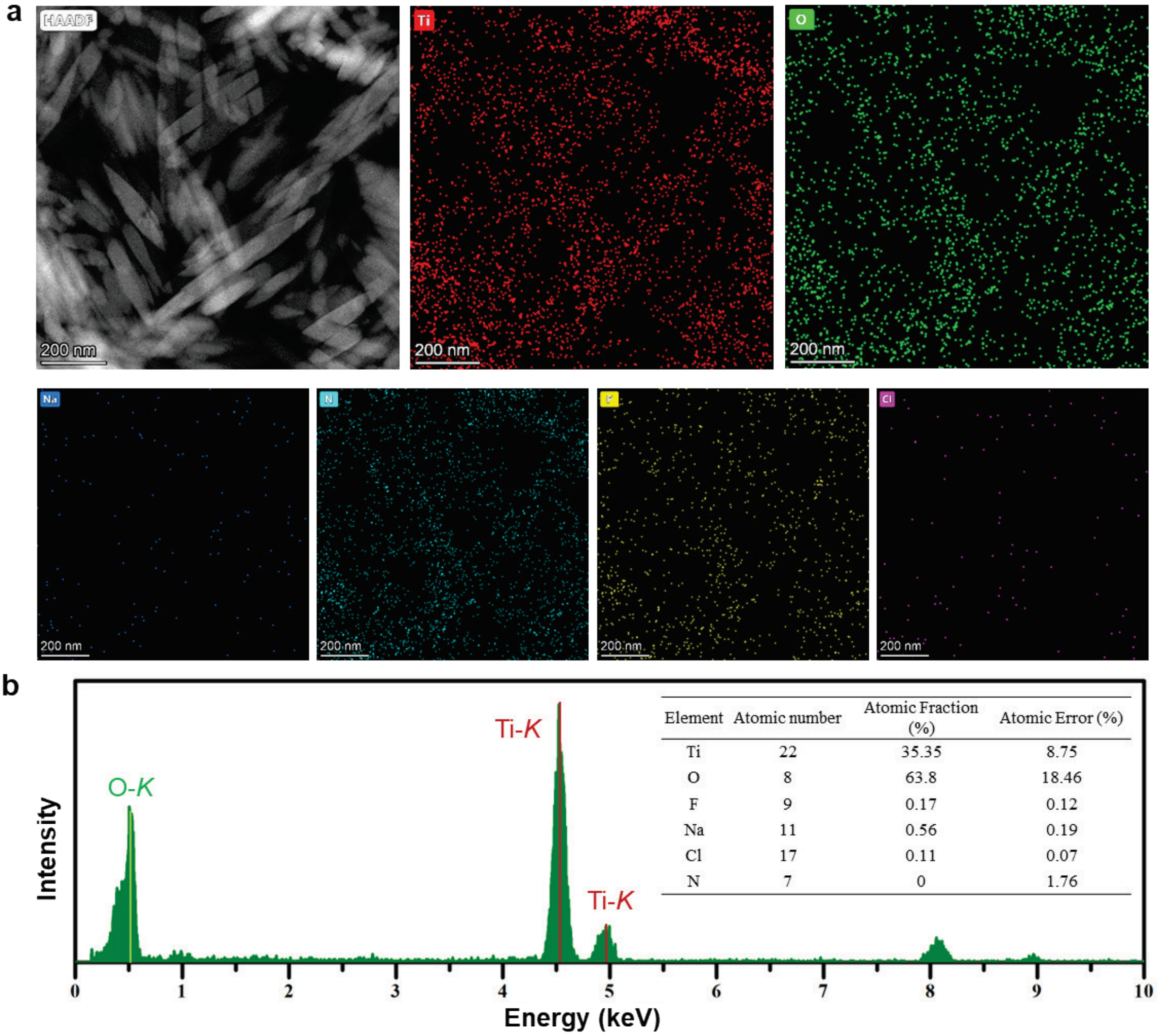

Element	Atomic number	Atomic Fraction (%)	Atomic Error (%)
Ti	22	35.35	8.75
O	8	63.8	18.46
F	9	0.17	0.12
Na	11	0.56	0.19
Cl	17	0.11	0.07
N	7	0	1.76

Supplementary Fig. 1. a HAADF-STEM and EDS elemental mapping of Ti and O elements, and possible influencing elements; **b** quantitative element diagrams of the prepared anatase nanorods from (**a**).

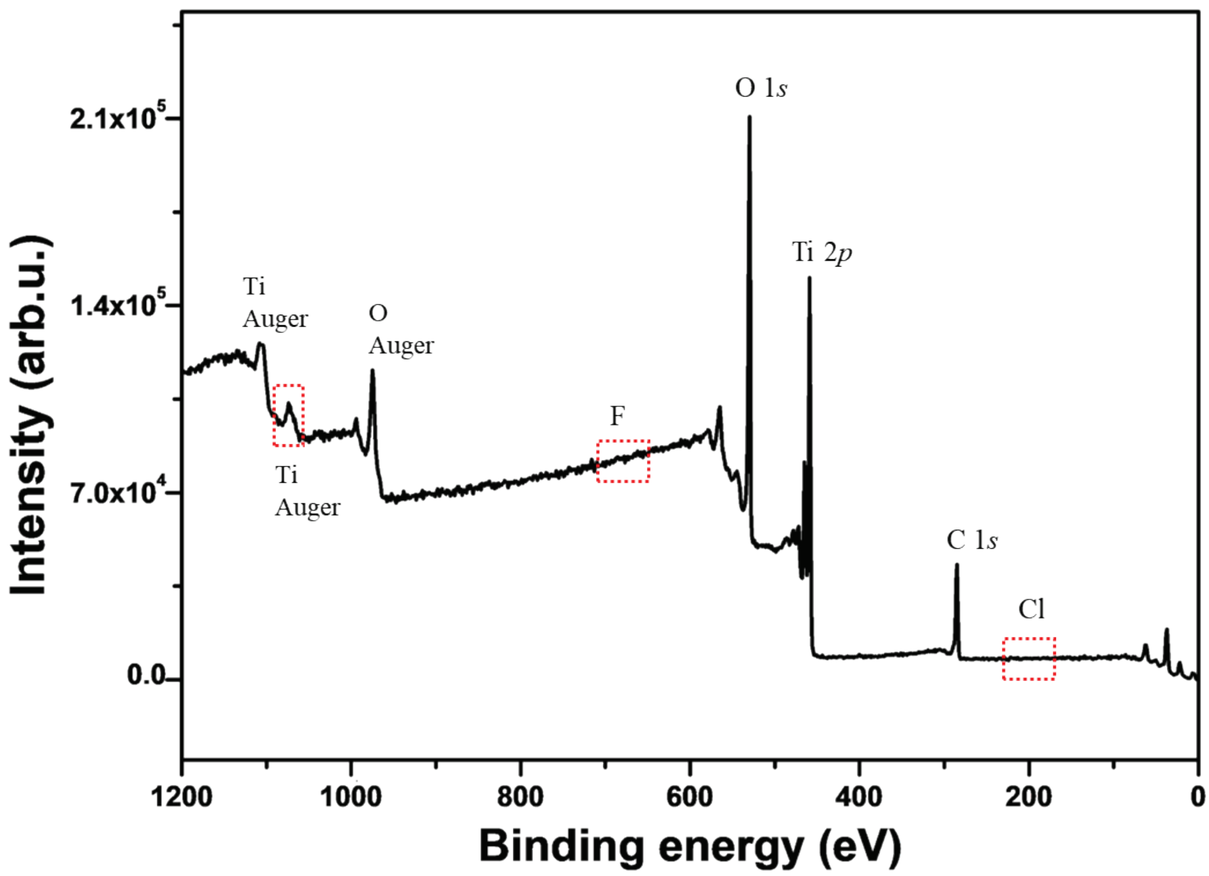

Supplementary Fig. 2. XPS spectra of the prepared anatase nanorods.

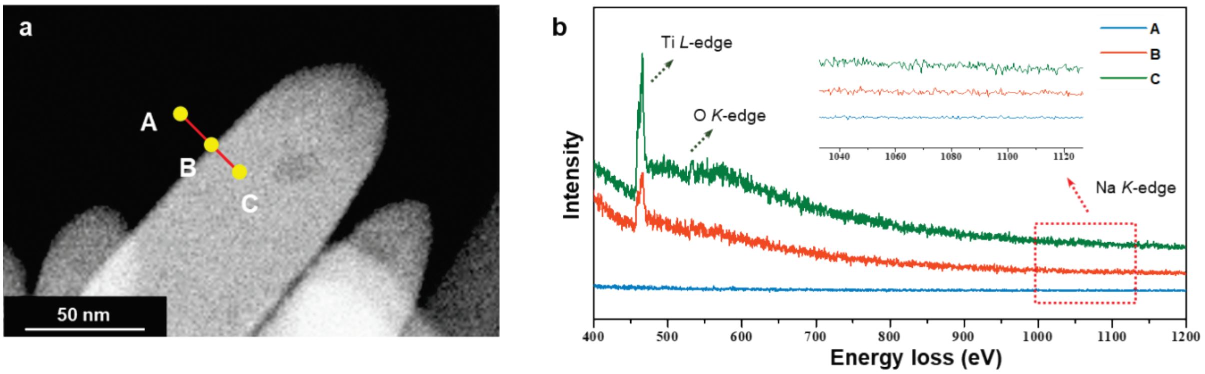

Supplementary Fig. 3. a ADF STEM image showing the anatase TiO_2 nanorod configuration under vacuum at room temperature with EB irradiation. **b** EELS spectra extracted from the yellow points in (**a**).

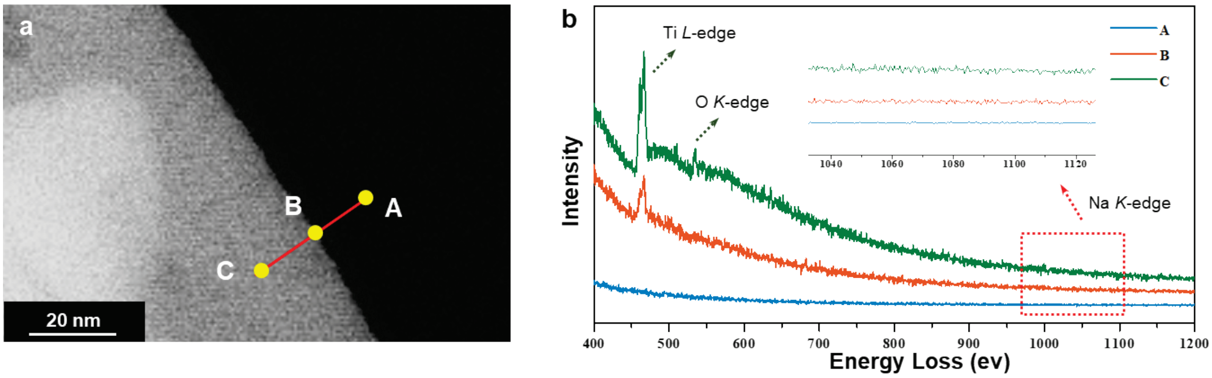

Supplementary Fig. 4. a ADF STEM image showing the anatase TiO_2 nanorod configuration under vacuum at room temperature with EB irradiation. **b** EELS spectra extracted from the yellow points in (**a**).

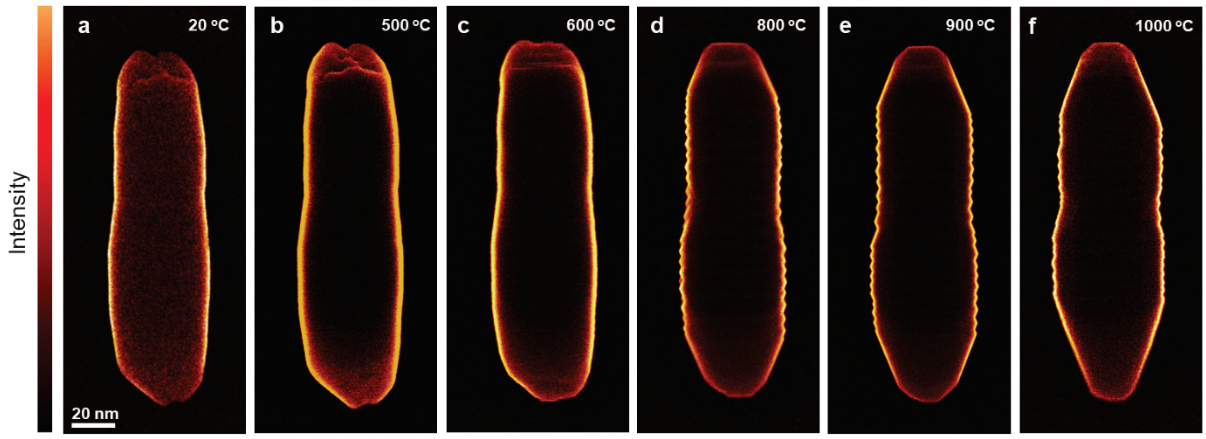

Supplementary Fig. 5. The morphological evolution of an anatase TiO_2 nanorod. In-situ HRSEM images of a typical anatase TiO_2 nanorod in the heating process in vacuum (TEM column pressure: 5×10^{-5} Pa). The heating rate is 1 °C s^{-1}, and each temperature should be maintained for a minimum duration of 30 min. **a-f** A typical morphological evolution process of the anatase TiO_2 nanorod at different temperatures. (**b–f**) share the same scale bar as (**a**). The color scale is linear.

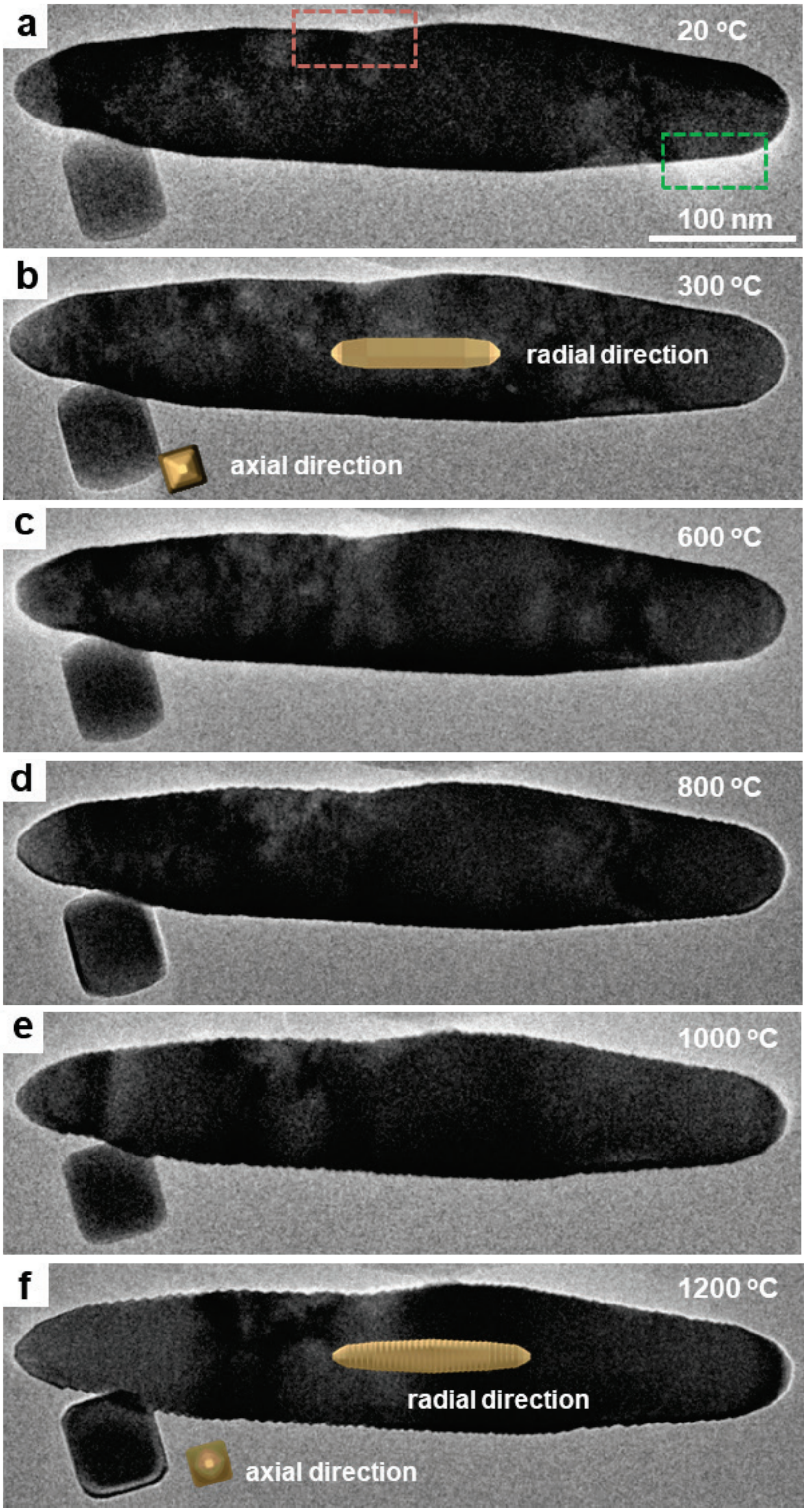

Supplementary Fig. 6. a-f In-situ TEM images from (Supplementary Movie 1) show the structure evolution of the typical anatase TiO_2 nanorods in the heating process in vacuum (temperature: 20-1200 °C; TEM column pressure: 5×10^{-5} Pa). The two TiO_2 nanorods were observed from two directions, both axial [001] and radial [010] viewing directions, respectively. (**b–f**) share the same scale bar as (**a**).

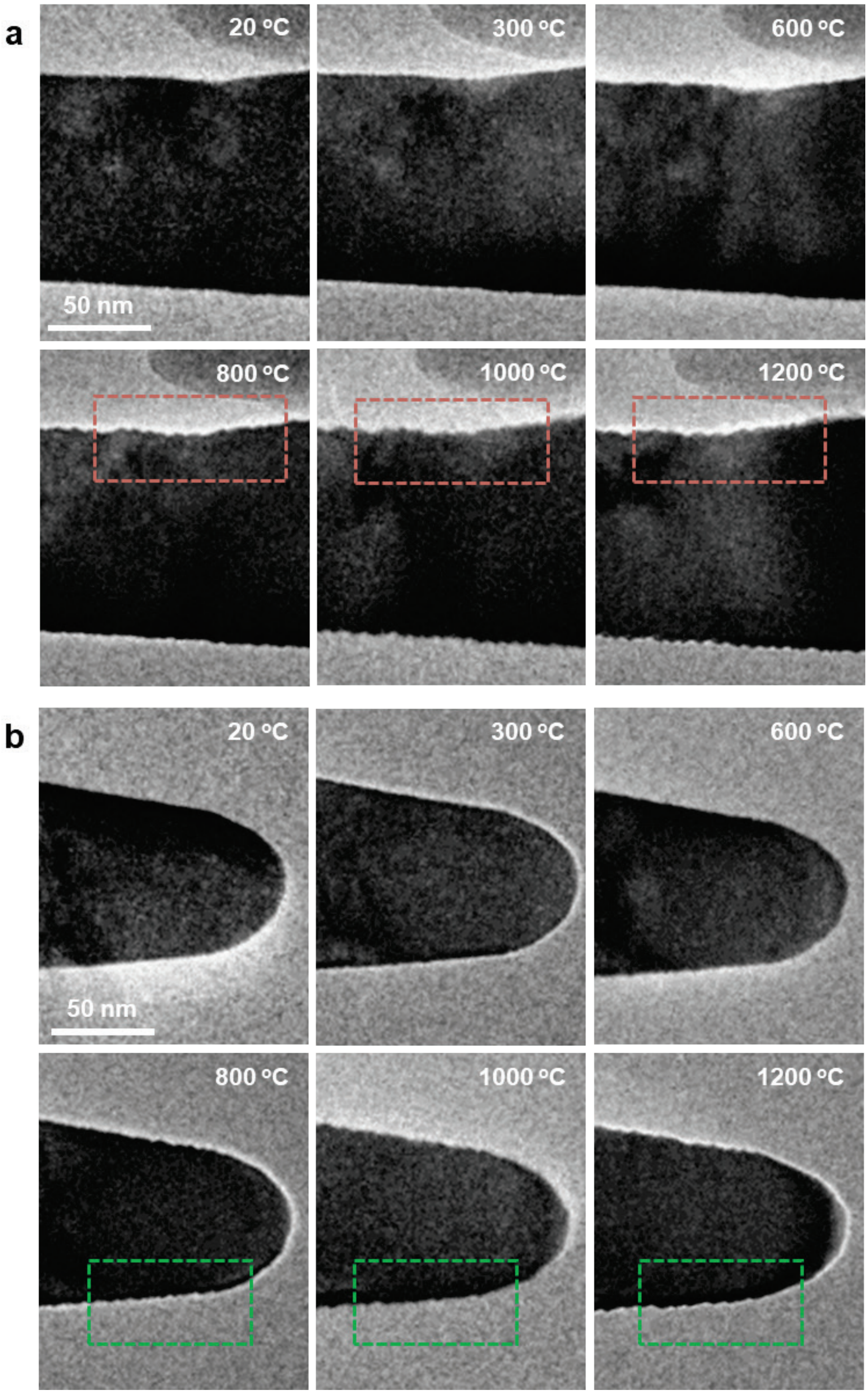

Supplementary Fig. 7. The enlarged in-situ TEM images show the structural evolution of the marked area (red box and green box areas) in Supplementary Fig. 6a a-b The images were collected in the heating process in vacuum (TEM column pressure: 5×10^{-5} Pa) show the at the corresponding temperatures. During the heating process, the originally flat (100) surfaces (**a**) and (301) surfaces (**b**) became roughened, reconstructing into distinct protruding structures. (**a–b**) share the same scale bar as (**a**).

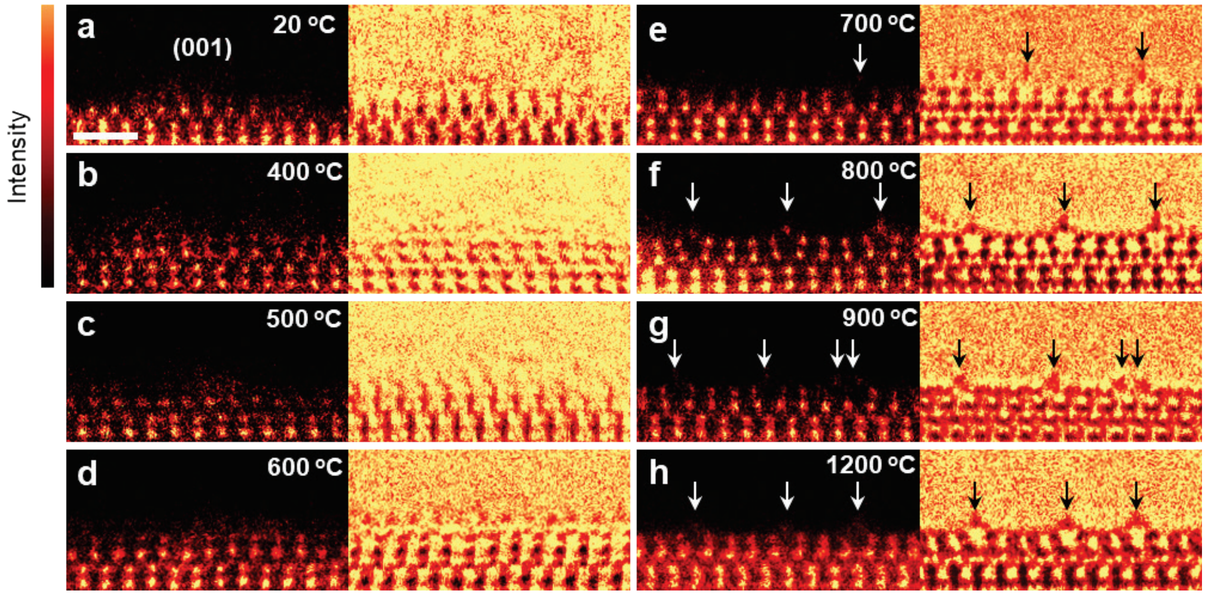

Supplementary Fig. 8. The atomic structural evolution of the anatase TiO_2 nanorod tip surface at the elevated temperatures. The heating rate is 1 °C s^{-1}, and each temperature should be maintained for a minimum duration of 30 min. **a-h** The atomic level in-situ HAADF STEM images show the structure evolution of (001) surface at the tip of anatase TiO_2 nanorod, in vacuum (temperature: 20-1200 °C; TEM column pressure: 5×10^{-5} Pa). Scale bar: 2nm. During the heating process, the defective (001) surface progressively gradually becomes unstable and begins to undergo reconstruction above 700 °C, and ultimately forms a (1×4) reconstruction structure at 1200 °C. (**b–h**) share the same scale bar as (**a**). The color scale is linear.

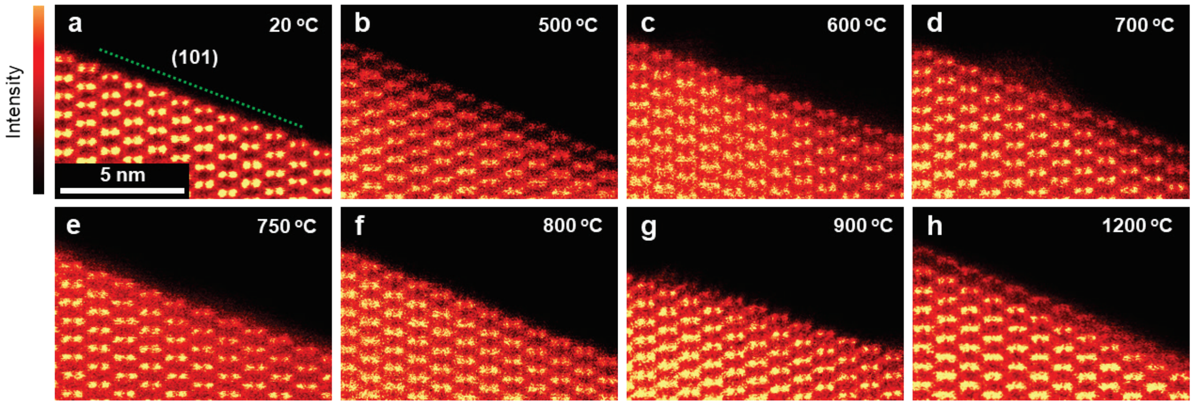

Supplementary Fig. 9. The atomic-level HAADF STEM images show the structure evolution of anatase TiO_2 (101) surface, in vacuum. (TEM column pressure: 5×10^{-5} Pa). The heating rate is 1 °C s^{-1}, and each temperature should be maintained for a minimum duration of 30 min. **a-h** The images are acquired at the corresponding temperatures. (**b–h**) share the same scale bar as (**a**). The color scale is linear.

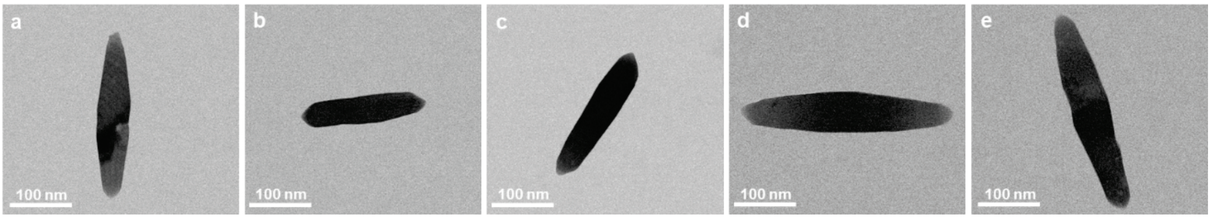

Supplementary Fig. 10. a-e BF images show the morphologies of five typical anatase TiO_2 nanorods at room temperatures in vacuum (column pressure: 10^{-5} Pa).

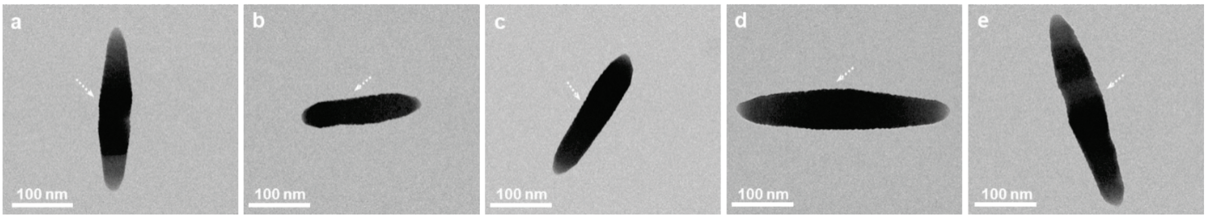

Supplementary Fig. 11 a-e BF images show the morphologies of five typical anatase TiO_2 nanorods at 1100 °C in vacuum (column pressure: 10^{-5} Pa) after being heated for more than 3 h. The morphological changes are consistent with those presented in Figure 1 of the main text, indicating that surface reconstruction of nanorods is a common and inevitable phenomenon throughout the experimental process.

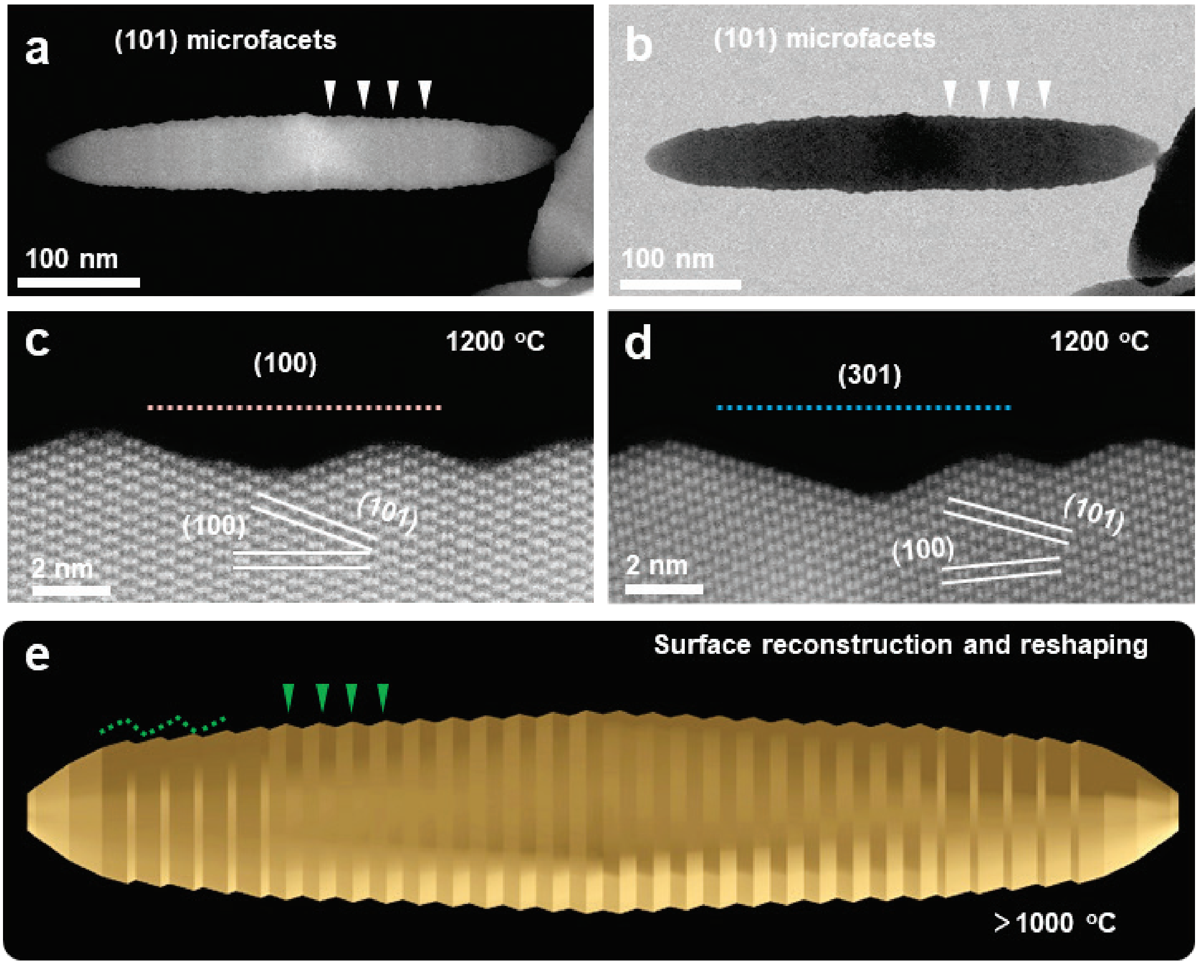

Supplementary Fig. 12 In-situ HAADF STEM images show the structural evolution of TiO_2 nanorod at high temperatures in vacuum (column pressure: 10^{-5} Pa), viewed along the [010] direction. a-b HAADF STEM (**a**) and BF (**b**) of morphology changes of a typical anatase TiO_2 nanorod after heating at 1200 °C for more than 5 h. **c-d** The atomic-level HAADF STEM images of (100) (**c**), (301) (**d**) surfaces at 1200 °C, after being heated for more than 5 h. The anatase (101) and (100) facets could be clearly identified, which confirm that the nanorod retains the anatase phase without transformation to rutile. **e** Schematic diagram of morphology changes of a typical anatase TiO_2 nanorod after heating.

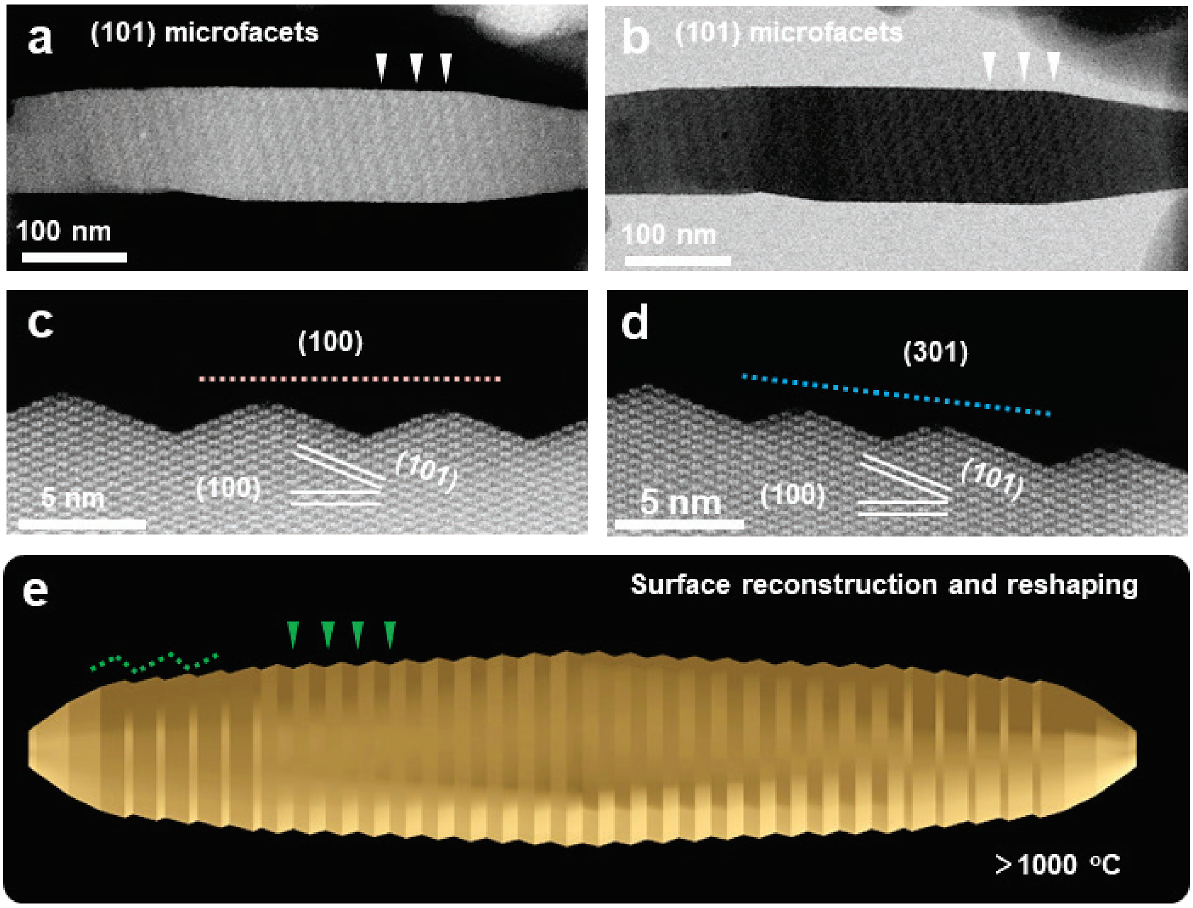

Supplementary Fig. 13 In-situ HAADF STEM images show the structural evolution of TiO_2 nanorod at high temperatures in vacuum (column pressure: 10^{-5} Pa), viewed along the [010] direction. a-b HAADF STEM (**a**) and BF (**b**) of morphology changes of a typical anatase TiO_2 nanorod after heating at 1100 °C for more than 5 h. **c-d** The atomic-level HAADF STEM images of (100) (**c**), (301) (**d**) surfaces at 1100 °C, after being heated for more than 5 h. The identified anatase (101) and (100) facets clearly confirm that the nanorod retains the anatase phase without transformation to rutile. **e** Schematic diagram of morphology changes of a typical anatase TiO_2 nanorod after heating.

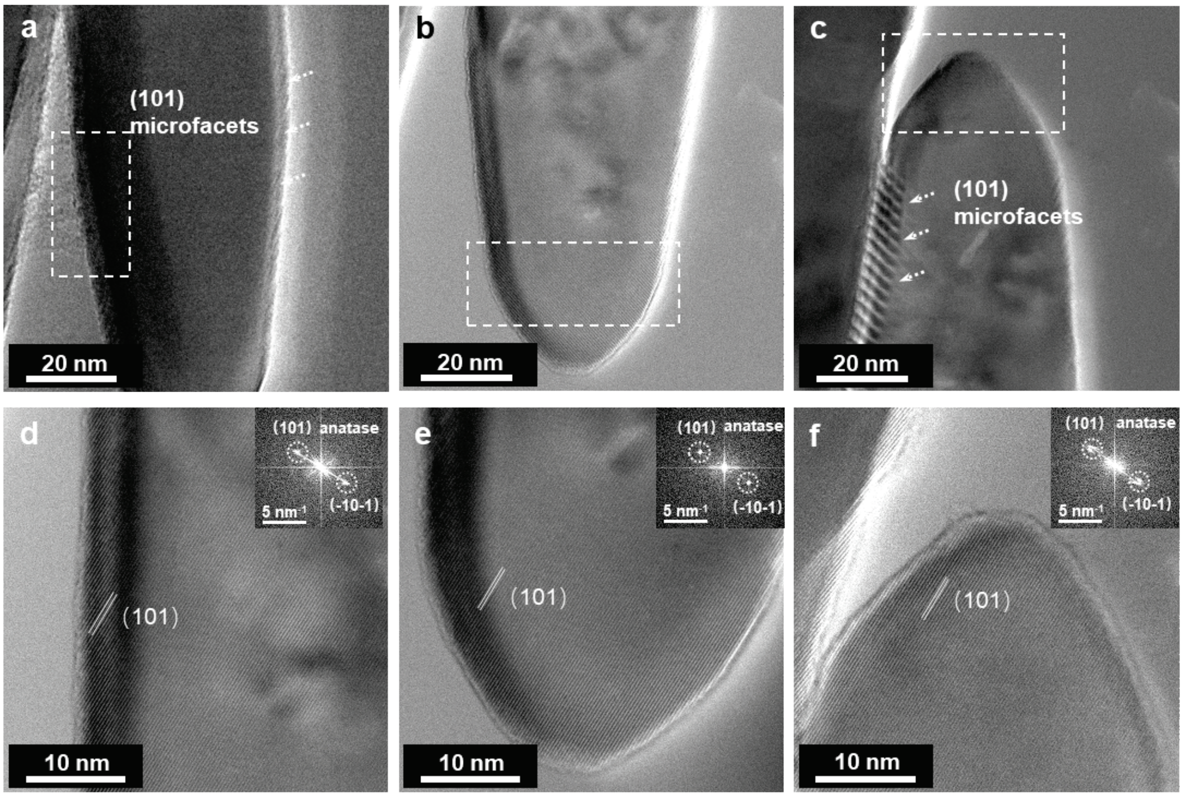

Supplementary Fig. 14 In-situ TEM images show the structural evolution of TiO_2 nanorod at high temperatures in vacuum (column pressure: 10^{-5} Pa), at 1100 °C after being heated for more than 10 h. a-c The middle (**a**), lower (**b**) and upper (**c**) parts of the same anatase TiO_2 nanorod, respectively. **d-f** The enlarged HRTEM image from the white dotted boxes in (**a-c**), the inserted images are the corresponding FFT patterns. From the HRTEM images and the corresponding FFT patterns, the anatase TiO_2 (101) facet could be clearly identified, which confirm that the nanorod retains the anatase phase without transformation to rutile.

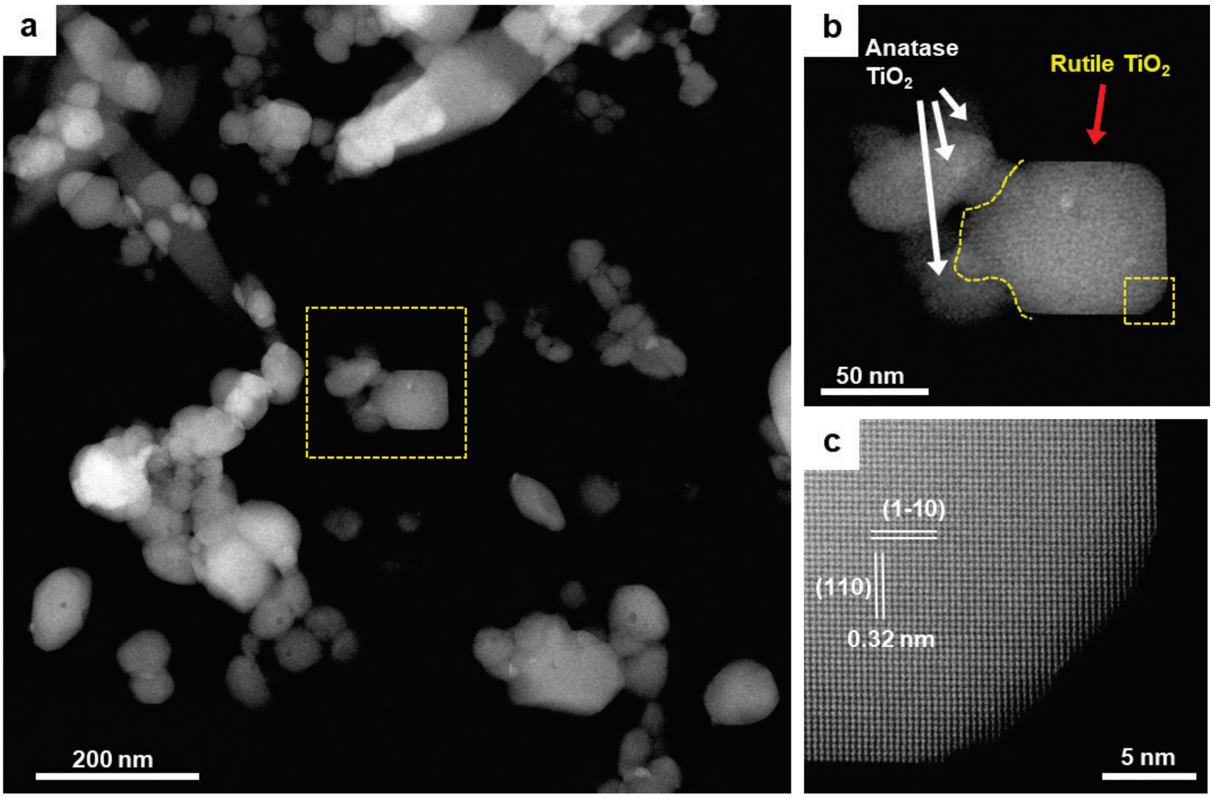

Supplementary Fig. 15 The in-situ HAADF STEM images show the nucleation of rutile TiO_2 crystallite (temperature: 1000 °C; TEM column pressure: 5×10^{-5} Pa). a-c The low magnification (**a-b**) and the atomic-level (**c**) HAADF STEM images show the rutile TiO_2 nucleated at the multi-grain boundaries by the convergence of adjacent anatase TiO_2 particles.

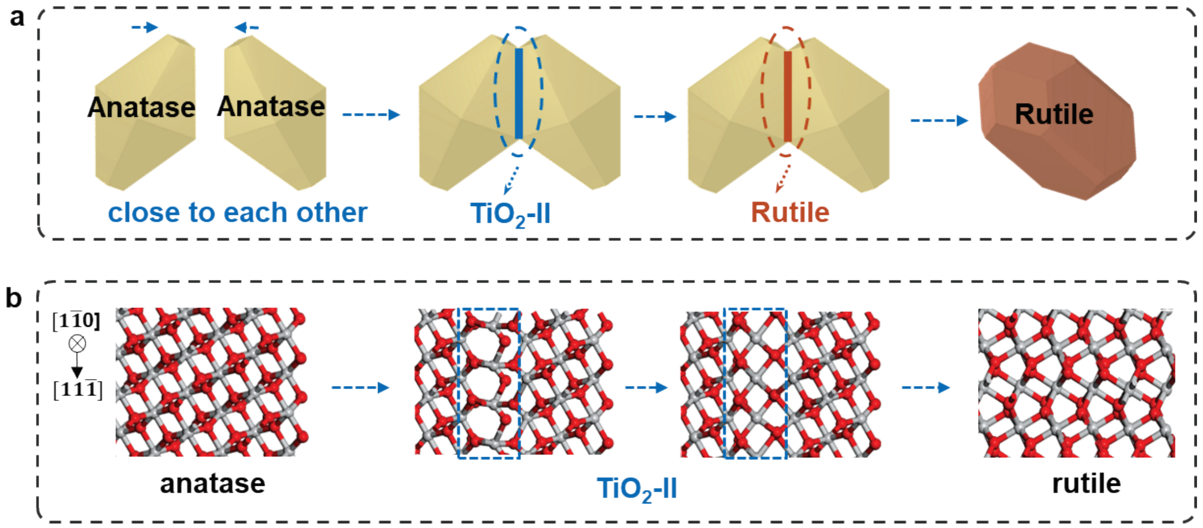

Supplementary Fig. 16 **a** Schematic diagram of 112 crystal plane formation and rutile nucleation. The models were created using CAD software. **b** Schematic diagram of phase transition from anatase to rutile. Red: O; Gray: Ti; Blue dashed box: Ti and O after reconstruction.
