# Supplementary material for: Atomic-level insights into the high intrinsic thermostability of individual anatase TiO2 nanocrystals through surface-locking effects
Source: Nat Commun. 2026 May 20;17:6658. doi: 10.1038/s41467-026-73332-5 (PMC13381851; doi:10.1038/s41467-026-73332-5)
Supplement: Supplementary file 2 — Description of Additional Supplementary Files [file 41467_2026_73332_MOESM2_ESM.pdf]

Description of Additional Supplementary Files

Supplementary Videos (1-3)

Captions of Videos

Video 1: In situ TEM video show the structure evolution of the typical anatase TiO_2 nanorods in the heating process in vacuum, viewed along both axial and radial directions. (Temperature: 20-1200 °C; TEM column pressure: 5×10^{-5} Pa).

Video 2: In situ atomic level STEM video show the surface structural evolution during the restructuring of a single crystalline TiO_2 nanorod, indicating a significant mass transportation via surface diffusion. The video collected at 1000 °C in vacuum (TEM column pressure: 5×10^{-5} Pa)

Video 3: In situ STEM video show the surface structural evolution of the sample in Video 1 at low magnification. The video collected at 1000 °C in vacuum (TEM column pressure: 5×10^{-5} Pa)
