# Supplementary material for: Atomic-level insights into the high intrinsic thermostability of individual anatase TiO2 nanocrystals through surface-locking effects
Source: Nat Commun. 2026 May 20;17:6658. doi: 10.1038/s41467-026-73332-5 (PMC13381851; doi:10.1038/s41467-026-73332-5)
Supplement: Supplementary file 6 — Transparent Peer Review file [file 41467_2026_73332_MOESM6_ESM.pdf]

nature portfolio

Peer Review File

Atomic-level insights into the high intrinsic thermostability of individual anatase TiO_2 nanocrystals through surface-locking effects

Corresponding Author: Professor Yong Wang

This file contains all reviewer reports in order by version, followed by all author rebuttals in order by version.

Version 0:

Reviewer comments:

Reviewer #1

(Remarks to the Author)
In the manuscript titled, "Unexpected Phase Stability of Anatase TiO2 Nanocrystals at Ultrahigh Temperature via Surface Restructuring," X. Guo et al. present STEM images of individual nanorods remaining in anatase after heating up to 1200 °C. The authors claim exceptional stability of anatase nanorods against heating and further attribute the phase stability to the so-called "surface locking" effect. However, this reviewer finds that the manuscript lacks much to substantiate their claim.

The anatase-to-rutile transition (ART) is a gradual, reconstructive transformation involving Ti diffusion, nucleation, and growth. The rate of transformation thus depends on temperatures, so does the ratio of transformed rutile to anatase on the duration of heating. For example, Gouma and Mills (JACS 2001) reported that a minimum heat treatment at 1000 °C for 8 hours was required for anatase nanoparticles to fully transform to rutile. More recently Lu et al. (PCCP 2023) reported that ART of micron-sized anatase crystals with a large percentage of (001) facet takes place after heating between 1050 and 1100 °C and the complete transformation takes up to 50 hours. Without information on the heating rate, the total duration of heating, and other specific procedures related to heating experiment for each sample discussed, their claims are uncorroborated.

It is well known that the ART occurs over a wide temperature range, e.g. between about 600 °C and 1200 °C, depending on many factors such as impurities, sample preparation methods, grain size, morphology, heating process, etc. The high sensitivity of the temperature onset for the phase transition has been extensively investigated in the past by R. D. Shannon (1964) and many other workers afterwards. In addition to the reports mentioned above on the measurable onset of ART exceeding 1000 °C, Krivtsov et al. also showed that the anatase nanoparticles synthesized by the solvent-exchange method are stable up to 1000 °C (RSC Advances 2015). So, what is the significance of this manuscript in claiming the thermal stability?

The authors made several statements referring to the high ART onset temperature, for example, "… maintain the anatase phase at 1250 °C, instead of turning into the rutile phase, which exceeded the reported anatase-to-rutile phase transition point nearly 650 °C higher" in Lines 31-33, similarly in Lines 91-93, 179-181, and 289-291. Without any references to the prior studies of inhibiting factors in ART, their statements are not just hyperbolic but misleading.

All the STEM observation of different surface restructuring and DFT calculations show are the relative energetics of low Miller-index surfaces of anatase – the surface energetics have been already well established. Are the authors claiming an "exceptional" stability due to the absence of (112) facets in the nanorods? There are neither analyses nor evidences presented in this manuscript that the absence/presence of such a controlling factor inhibits/promotes ART.

In summary, the manuscript is unsuitable for the publication of Nature Communication.

Reviewer #2

(Remarks to the Author)
The authors report on high thermal stability of individual anatase TiO_2 nanorod single crystals with detailed characterisation using different electron microscopy methods. They observe a noteworthy stability at 1250 °C, and give convincing reasoning behind for the observed stability of the individual crystals. The data analysis and methodology are sound for the most part, with the exception of elemental analysis of the TiO_2 nanorods missing from the manuscript. Even though the results are convincing and relatively interesting, I do not believe this manuscript reports results that are significant enough to the related fields to merit publication in Nature Communications. I instead recommend publishing the results in a more specialised journal.

I elaborate on my reasoning here:

1) The authors claim many times that they observe stability at temperatures exceeding typical anatase by 650 °C. However, this is a misleading claim since it only applies to pure bulk anatase. Anatase stability has been studied for more than 60 years in the literature, and the stability of the anatase phase is well known to depend greatly on impurities, the synthetic procedure, and the atmosphere during heat treatment. The reported values for anatase-to-rutile transformation indeed vary between 400–1200 °C in the literature (e.g. 10.1007/s10853-010-5113-0). The authors utilise a synthesis with Na-titanate precursors, and TiO_2 with Na_2O impurities have been long ago reported to show a phase transition temperature exceeding 900 °C (10.1111/j.1151-2916.1961.tb13725.x). The fact that the individual nanocrystals in the manuscript show stability exceeding 1250 °C is not surprising, considering that anatase samples have been shown to remain stable at up to 1200 °C (10.1111/j.1151-2916.1965.tb14774.x).

2) The fact that the annealing in the manuscript is performed in vacuo also affects the phase transformation, with reports spanning back 60 years how titanium interstitials retard the phase transformation due to a "structure stuffing effect" (10.1111/j.1151-2916.1965.tb14774.x). This is to my understanding the same process the authors call "surface locking".

3) The reconstruction of TiO_2 nanocrystal to a majority of (101) surface instead of (112) surface is not surprising, and was published by many of the same authors 10 years ago (10.1021/acs.nanolett.5b03277).
Furthermore, the importance of the (112) surface in the anatase-to-rutile transformation has been known for more than 20 years (10.1111/j.1151-2916.2001.tb00709.x). I agree with the authors assignment for reasons behind the retardation of the phase transformation, but do not think the results are particularly significant to a wide audience.

Reviewer #3

(Remarks to the Author)
The experimental results of this manuscript are quite beautiful and instructive. Still, their presentation needs major revision: It is absolutely untrue that anatase is not stable in microscopic form -- only for nanomaterials, it converts to rutile irreversibly. Surface science work (ignored completely here) has clearly shown that antatase (101) single crystals are very stable, up to highest temperature. This is different from brookite, where the temperature-driven phase transition is indeed occuring at several hundred °C.

What the authors observe - in a very nice and detailed fashion is just the restructuring of high-energy surfaces to the lowest-energy one, the 101 surface. It has been known since more than 25 years (see DFT calculations of the Wulff constrution by Selloni, for example), that this surface is exceedingly stable. It is no wonder, then, - but still nice to see - that the relatively big 'nanograins' of anatase show faceting of their high-energy surfaces to 101 facets. Also, a well known and entirely expected phenomenon based on decades of surface science research.

Because many statements are ignoring prior knowledge on this system they are misleading. The good news is that, in my opinion, the experiments do not need to be repeated or extended -- just re-interpreted in the proper way.

Without these major revisions, I cannot recommend publication of this paper.

Version 1:

Reviewer comments:

Reviewer #1

(Remarks to the Author)
The manuscript titled, “Excellent Phase Stability of Individual Anatase TiO_2 Nanocrystals via Reshaping and Surface Restructuring,” by X. Guo et al. has been reviewed.

This revised manuscript focuses on the mechanism of phase stability in individual anatase TiO_2 nanorods. Using in situ atomic-resolution STEM, the authors show that high-temperature stability arises from surface restructuring, where facets evolve into low-energy (101) surfaces, creating a “surface-locking” effect that kinetically inhibits rutile nucleation. In contrast, aggregated nanorods transform readily, highlighting the role of surface dynamics in phase stability.

However, the central observation and interpretation largely confirm what is already well established. It is known that among low-index anatase surfaces, only the (112) facet provides nucleation sites for the anatase-to-rutile transition (Zhu et al., J.

Am. Chem. Soc. 137, 11532 (2015)). Restructuring of (101) and (100) surfaces costs ~0.71 eV and ~0.82 eV, respectively, compared to ~0.45 eV for (112), and only (112) surfaces allow restructuring to propagate into the bulk. Therefore, in the absence of (112) facets, isolated nanorods are expected to remain anatase. The STEM observations and DFT calculations presented here essentially validate this known model rather than providing fundamentally new insights.

In summary, while the experiments are carefully executed, the mechanistic conclusions do not advance the current understanding sufficiently for publication in Nature Communications. The work would be more appropriate for a specialized materials science journal.

Reviewer #2

(Remarks to the Author)
The authors report on high thermal stability of individual anatase TiO_2 nanorod single crystals with detailed characterisation using different electron microscopy methods. The authors have revised their manuscript by modifying the discussion (albeit only slightly) and performing elemental analysis of their nanoparticles. The elemental analysis gives required information on the composition of the nanoparticles. Even with the added analysis and corrections to the text, previously incorrect statements remain, such as "The phase structure of anatase TiO_2 could transformed into rutile around ~600 °C", which is not an apples-to-apples comparison to the individual nanoparticles here. They have for the most part added the references I required, but the amount of discussion on the importance of their results relative to the existing literature remains lacking. Even though the measurements are quite interesting, I do not see a reason to change my previous assessment that the results in this manuscript are not significant enough to the related fields to merit publication in Nature Communications. I instead recommend publishing the results in a more specialised journal.

Reviewer #3

(Remarks to the Author)
The manuscript has been improved; it no longer overlooks long-established concepts regarding the surface energy of nanoparticles in the anatase phase. It is a nice confirmation of these concepts and indeed interesting to specialists. It is not of appropriate generality and impact for a publication in Nature Communications, however.

Open Access This Peer Review File is licensed under a Creative Commons Attribution 4.0 International License, which permits use, sharing, adaptation, distribution and reproduction in any medium or format, as long as you give appropriate credit to the original author(s) and the source, provide a link to the Creative Commons license, and indicate if changes were made.
In cases where reviewers are anonymous, credit should be given to 'Anonymous Referee' and the source.
The images or other third party material in this Peer Review File are included in the article's Creative Commons license, unless indicated otherwise in a credit line to the material. If material is not included in the article's Creative Commons license and your intended use is not permitted by statutory regulation or exceeds the permitted use, you will need to obtain permission directly from the copyright holder.
To view a copy of this license, visit https://creativecommons.org/licenses/by/4.0/

Point-by-point response to Reviewers

Reviewer #1:

General comments: In the manuscript titled, "Unexpected Phase Stability of Anatase TiO_2 Nanocrystals at Ultrahigh Temperature via Surface Restructuring," X. Guo et al. present STEM images of individual nanorods remaining in anatase after heating up to 1200 °C. The authors claim exceptional stability of anatase nanorods against heating and further attribute the phase stability to the so-called "surface locking" effect. However, this reviewer finds that the manuscript lacks much to substantiate their claim.

Response: Thank you for the constructive and insightful comments, which has been instrumental in enhancing the quality of our work. Per your comments, we have made substantial revisions to our manuscript, and we are confident that we have fully addressed all your concerns.

Comment #1: The anatase-to-rutile transition (ART) is a gradual, reconstructive transformation involving Ti diffusion, nucleation, and growth. The rate of transformation thus depends on temperatures, so does the ratio of transformed rutile to anatase on the duration of heating. For example, Gouma and Mills (JACS 2001) reported that a minimum heat treatment at 1000 °C for 8 hours was required for anatase nanoparticles to fully transform to rutile. More recently Lu et al. (PCCP 2023) reported that ART of micron-sized anatase crystals with a large percentage of (001) facet takes place after heating between 1050 and 1100 °C and the complete transformation takes up to 50 hours. Without information on the heating rate, the total duration of heating, and other specific procedures related to heating experiment for each sample discussed, their claims are uncorroborated.

Response: Thank you for the insightful comments and provide us important references. We agree with you that the ART is a gradual, reconstructive transformation which strongly depends on temperatures. Following the reviewer's suggestion, the detailed information concerning the heating rate, the duration, etc., for the experiments has been added to the updated manuscript for a comprehensive discussion. Beyond addressing these experimental details, we would like to emphasize that the key finding of our work lies in the remarkable difference in thermal stability observed between individual single-crystal nanorods and their aggregated

counterparts under identical thermal treatment conditions. Specifically, our conclusions are well supported by the following reasons:

(1) In a direct comparison under identical thermal treatment, aggregated nanorods underwent complete and rapid transformation to rutile within several minutes, while well-dispersed individual nanorods remained in the anatase phase, even with a longer duration of heating (several hours). This stark difference underscores that the aggregation state—individual versus overlapping—is highly relevant to the transformation. Our results consistently support a contact-induced nucleation mechanism [*J. Am. Chem. Soc. 2015, 137, 35, 11532–11539*]. The phase transition necessitates the nucleation at (112) twin boundaries, which could be achieved through specific oriented contact between distinct particles. This clearly explains the high stability of individual nanorods against transformation into the rutile phase, primarily due to the absence of suitable nucleation sites such as (112) twin boundaries. After reconstruction, the surfaces are fully reconstructed into thermodynamically stable (101) and (1×4)-(001) facets, thereby effectively suppressing nucleation. Therefore, the facile phase transformation in multiple overlapping nanorods can be attributed to the fact that their mutual contact provides a statistical probability of forming (112) twin boundaries, which in turn serve as effective nucleation sites for the rutile phase.

(2) Although the effects of heating temperature and duration are not the primary focus of this discussion, we conducted additional experiments informed by the referenced literature to evaluate the thermal stability of individual nanorods under more extreme conditions. From the new experiments, it is evident that even under more extreme conditions (e.g. temperature: 1100 °C, duration＞10 h), comparable to those used in previously reported studies [*J. Am. Ceram. Soc. 2001, 84, 619–622; Phys. Chem. Chem. Phys. 2023, 25, 3199-3210*], the individual single-crystalline anatase nanorod retain the anatase phase, despite undergoing significant mass transport accompanied by morphological changes and surface atomic reconstruction (Figures R1~R5). This indicates the high phase stability of individual anatase TiO_2 nanocrystal is not due to limitations in mass transfer, insufficient temperature or duration, but rather stems from the constraints inherent to the nucleation process. At this temperature, mass transport via diffusion becomes highly facile, as evidenced by the morphological and surface atomic restructuring observed in our study (Figures. 1~5 in manuscript and Video 1~3).

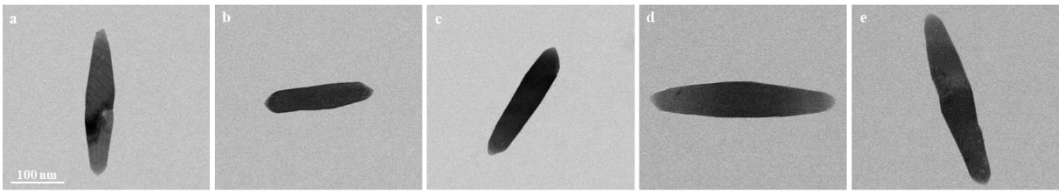

Fig. R1. (Fig. 11 in SI) a-e, BF images show the morphologies of five typical anatase TiO_2 nanorods at room temperatures in vacuum (Column pressure: 10^{-5} Pa).

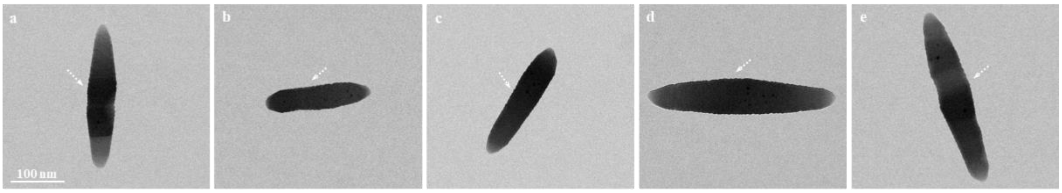

Fig. R2. (Fig. 12 in SI) a-e, BF images show the morphologies of the five anatase TiO_2 nanorods at 1100 °C in vacuum (Column pressure: 10^{-5} Pa), after being heated for more than 3 hours. The morphological changes are consistent with those presented in Figure 1 of the main text, indicating that surface reconstruction of nanorods is a common and inevitable phenomenon throughout the experimental process.

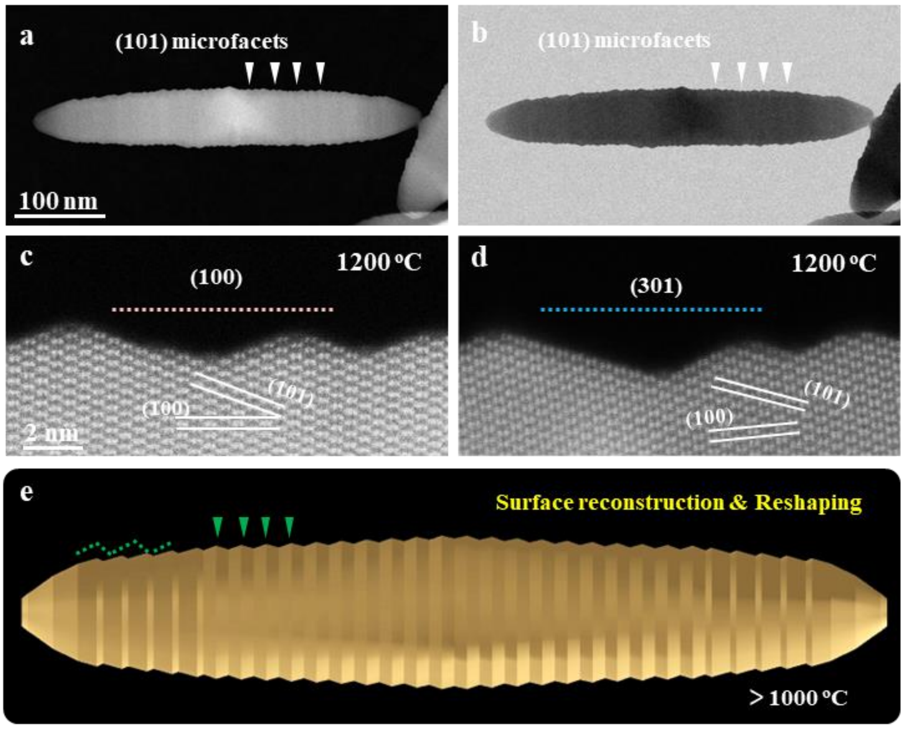

Fig. R3. (Fig. 13 in SI) In situ HAADF STEM images show the structural evolution of TiO_2 nanorod at high temperatures in vacuum (Column pressure: 10^{-5} Pa), viewed along the [010] direction. a-b,

HAADF STEM (a) and BF (b) of morphology changes of a typical anatase TiO_2 nanorod after heating at 1200 ° C for more than 5 hours. **c-d,** The atomic level HAADF STEM images of (100) (c), (301) (d) surfaces at 1200 ° C, after being heated for more than 5 hours. The anatase (101) and (100) facets could be clearly identified, which confirm that the nanorod retains the anatase phase without transformation to rutile. **e,** Schematic diagram of morphology changes of a typical anatase TiO_2 nanorod after heating.

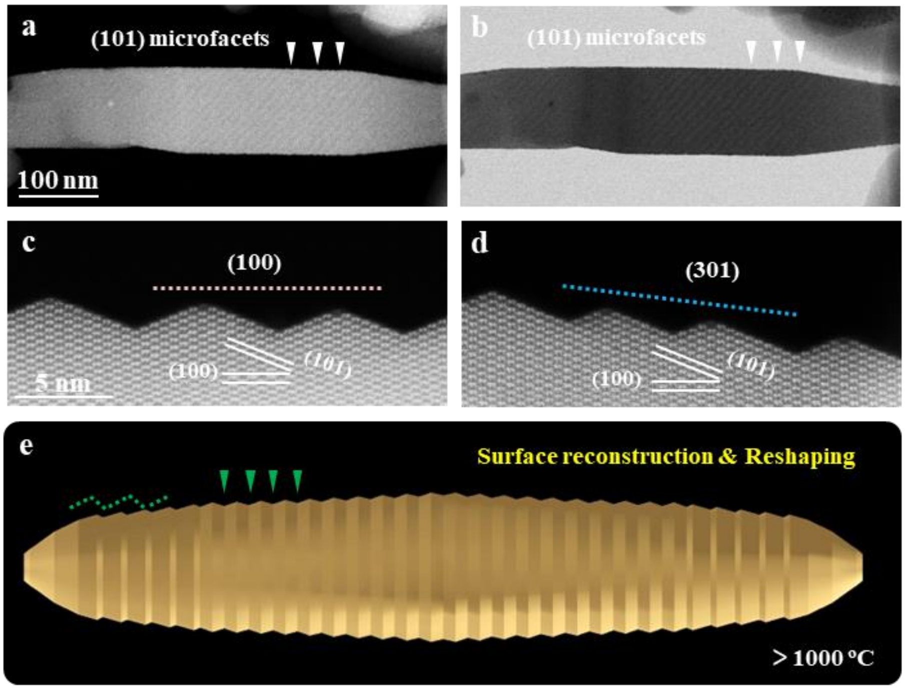

Fig. R4. (Fig. 14 in SI) In situ HAADF STEM images show the structural evolution of TiO_2 nanorod at high temperatures in vacuum (Column pressure: 10^{-5} Pa), viewed along the [010] direction. a-b, HAADF STEM (a) and BF (b) of morphology changes of a typical anatase TiO_2 nanorod after heating at 1100 ° C for more than 5 hours. **c-d,** The atomic level HAADF STEM images of (100) (c), (301) (d) surfaces at 1100 ° C, after being heated for more than 5 hours. The identified anatase (101) and (100) facets clearly confirm that the nanorod retains the anatase phase without transformation to rutile. **e,** Schematic diagram of morphology changes of a typical anatase TiO_2 nanorod after heating.

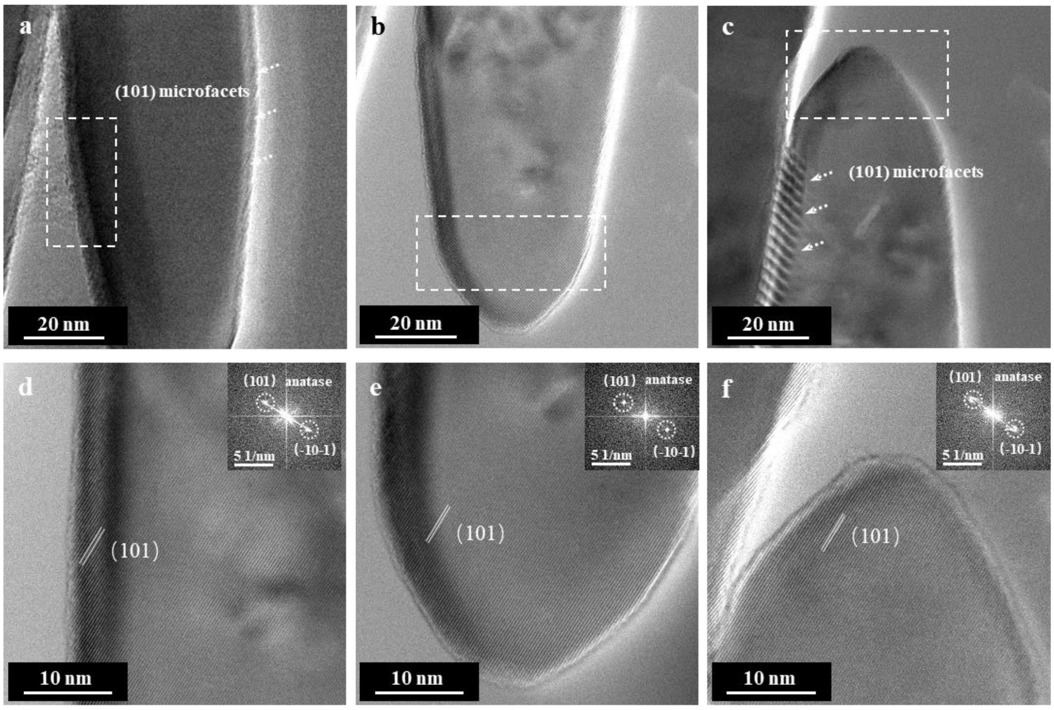

Fig. R5. (Fig. 15 in SI) In situ TEM images show the structural evolution of TiO_2 nanorod at high temperatures in vacuum (Column pressure: 10^{-5} Pa), at 1100 ° C after being heated for more than 10 hours. a-c, The middle (a), lower (b) and upper (c) parts of the same anatase TiO_2 nanorod, respectively. **d-f,** The enlarged HRTEM image from the white dotted boxes in (a-c), the inserted images are the corresponding FFT patterns. From the HRTEM images and the corresponding FFT patterns, the anatase TiO_2 (101) facet could be clearly identified, which confirm that the nanorod retains the anatase phase without transformation to rutile.

Comment #2: It is well known that the ART occurs over a wide temperature range, e.g. between about 600 °C and 1200 °C, depending on many factors such as impurities, sample preparation methods, grain size, morphology, heating process, etc. The high sensitivity of the temperature onset for the phase transition has been extensively investigated in the past by R. D. Shannon (1964) and many other workers afterwards. In addition to the reports mentioned above on the measurable onset of ART exceeding 1000 °C, Krivtsov et al. also showed that the anatase nanoparticles synthesized by the solvent-exchange method are stable up to 1000 °C (RSC Advances 2015). So, what is the significance of this manuscript in claiming the thermal stability?

Response: Thank you for your constructive comments. We agree with you that the phase transition points of anatase-to-rutile have been reported in a wide range, in different experimental measurements across different studies, and that the variability was attributed to numerous factors including impurities, synthesis methods, and atmosphere, etc. Actually, the broad transition temperature range reported in the literature precisely underscores the fact that the fundamental factors controlling anatase stability are not yet fully understood. Although some general conclusions have been drawn, how these factors, such as surface effect govern the thermal stability of TiO_2 remains unclear.

To bridge this gap, our study provides unprecedented in situ, atomic-level insight into this classic problem, through a state-of-the-art spherical aberration-corrected environmental scanning transmission electron microscope, which equipped with a high-resolution secondary electron detector for surface imaging. By in situ investigating a well-designed model system—single-crystal nanorods with uniform dimensions, well-defined morphology, and high crystallinity—we have elucidated the intrinsic phase stability of individual nanorods at the atomic scale. Through a direct comparison between isolated nanorods and their overlapping aggregates, we have confirmed the key factor of inter-particle contact for nucleation. Our results reveal that the surface reconstruction could also exert a profound impact on phase stability, as it significantly enhances stability by effectively suppressing surface nucleation in individual nanoparticles. Specifically, our work provides new insights in the following aspects:

(1) **Providing in situ atomic-scale insight:** Previous works have also investigated the influence of different factors on phase transitions, but mainly based on ex-situ characterization and theoretical calculations, lacking clear atomic-scale in-situ evidence. Previous investigations concerning the phase stability, predominantly relying on post-mortem characterization or theoretical simulations, lack the spatial and temporal resolution to capture dynamic surface restructuring processes. In this work, we integrated the state-of-the-art techniques, Cs-corrected STEM and atomic-scale secondary electron imaging, to elucidate the underlying mechanism within a well designed model system. Our work bridges the knowledge gap by delivering unprecedented in situ atomic-resolution visualization of surface and interfacial dynamics during ART.

(2) **Revealing intrinsic stability of individual nanocrystal:** Although some general conclusions have been drawn from previous ex situ studies, the essential factors governing its thermal stability remain elusive, and experimental interpretation in many previous reports are also complicated. In previous

studies, samples often differed considerably across experiments in terms of defects, dopants, morphology, and microstructure. The concurrent influence of these varied factors has made it challenging to accurately evaluate the intrinsic stability of individual nanocrystals. By utilizing high-purity single-crystal nanorods with uniform dimensions and well-defined faceting, we have created a model system that effectively decouples surface effects from other variables. This well-defined model system allows us to accurately evaluate the specific contribution of surface restructuring to phase stability, by excluding influences from structural heterogeneity, chemical doping, and environmental variations, etc.

(3) **Elucidating the surface effects in phase stability:** The anatase-to-rutile transition could be influenced by several structural factors, such as doping, defects, etc. For instance, dopants or defects can either enhance or impede Ti-atom mobility, thereby correspondingly decreasing or increasing phase stability. However, although factors such as structural heterogeneity, chemical doping, and environmental variations have been extensively investigated, the specific influence of intrinsic surface effects on phase stability has rarely been systematically addressed. Furthermore, a fundamental question remains unresolved: whether the phase transition is governed primarily by mass transport limitations or by nucleation barriers. Through a direct comparison between isolated nanorods and their overlapping aggregates, we have confirmed that nucleation at the interparticle contact interface is an essential step in the phase transition. Our results also reveal that the surface reconstruction could also exert a profound impact on phase stability, as it significantly enhances stability by effectively suppressing surface nucleation in individual nanoparticles.

(4) **Proposing “surface locking” mechanism:** Our work establishes that surface restructuring plays a dominant role in stabilizing anatase TiO_2 nanocrystals under high-temperature conditions. We discovered that dynamic surface reconstruction not only lowers the total energy of the system but also kinetically inhibits rutile nucleation. This finding provides atomic-scale insight into how surface structure and its evolution critically govern phase stability in nanomaterials. The proposed "surface locking" mechanism, validated through direct in situ observation, represents a fundamental advance and offers a generalizable principle for designing high-temperature functional nanomaterials through controlled surface engineering.

Comment #3: The authors made several statements referring to the high ART onset temperature, for example, "… maintain the anatase phase at 1250 °C, instead of turning into the rutile phase, which exceeded the reported anatase-to-rutile phase transition point nearly 650 °C higher" in Lines 31-33, similarly in Lines 91-93, 179-181, and 289-291. Without any references to the prior studies of inhibiting factors in ART, their statements are not just hyperbolic but misleading.

Response: We sincerely thank the reviewer for this critical comment and for pointing out the lack of precision in our original statements regarding the phase transition temperature. We apologize that the statement of the phase transition temperature in our previous manuscript was indeed potentially misleading, as you rightly pointed out. Following your advice, (1) we have thoroughly revised the relevant descriptions in the manuscript to make the expression more rigorous. (2) We have also supplemented relevant references and cited the prior studies of inhibiting factors in ART. The focus of manuscript is no longer on overstating the specific temperature value. Instead, we now emphasize the atomic-scale surface evolution process revealed by our in situ observations, and the striking difference in phase-transition behavior between individual and aggregated nanorods under identical conditions. (3) In addition, We have performed supplementary experiments, which allowed us to exclude the influence of other possible factors inhibiting the ART.

ART occurs over a wide temperature range, depending on many factors. Previous works have also investigated the influence of different factors on phase transitions, such as dopants, particle size, and atmosphere (*J. Mater. Sci. 2011, 46, 855 –874; J. Am. Chem. Soc. 2005, 127, 8659 –8666; J. Eur. Ceram. Soc. 2006, 9, 1527-1534; J. Am. Ceram. Soc. 1961, 44, 120-127*). We have added the discussions of the relevant factors to the manuscript. In our experiments, we integrated spherical aberration corrected environmental scanning transmission electron microscope, atomic-scale secondary electron surface imaging, and the construction of a highly regular model system, to clarify the critical role of the surface in ART process. By in situ investigating a well-designed model system—single-crystal nanorods with uniform dimensions, well-defined morphology, and high crystallinity—we have elucidated the intrinsic phase stability of individual nanorods at the atomic scale. Through a direct comparison between isolated nanorods and their overlapping aggregates, we have confirmed that nucleation at the interparticle contact interface is an essential step in the phase transition. Our results also reveal that surface reconstruction could also exert a profound

impact on phase stability by effectively suppressing surface nucleation, which enables the material to maintain remarkable stability even at 1250 ° C.

Furthermore, we have systematically ruled out the potential stabilizing effect of residual sodium from our Na-titanate precursor. In this work, since Na-titanate was used as the precursor, several measures were taken to exclude any consequential influence of sodium. The synthesized nanorods were first thoroughly washed with deionized water to remove any potential surface residues, which is a standard procedure for removing surface contaminants (*Sci. Sin. Chim. 2014, 44,1618; Sci Rep. 2015, 5, 12143*). Subsequently, Energy-Dispersive X-ray Spectroscopy (EDS), X-ray Photoelectron Spectroscopy (XPS), and Electron Energy Loss Spectroscopy (EELS) analyses (Figures R6~R9) were conducted to determine the presence of residual sodium. In both EDS, XPS and EELS data, no obvious Na signals were observed (Figures R6~R9), indicating that they did not play a significant role in our study (no distinct signal corresponding to the Na element was observed within its characteristic energy range (1.04 – 1.06 keV) in the EDS analysis, and no discernible signal was detected at the expected energy position for Na (1072 eV) in the EELS data), which have been added in the Supplementary Information.

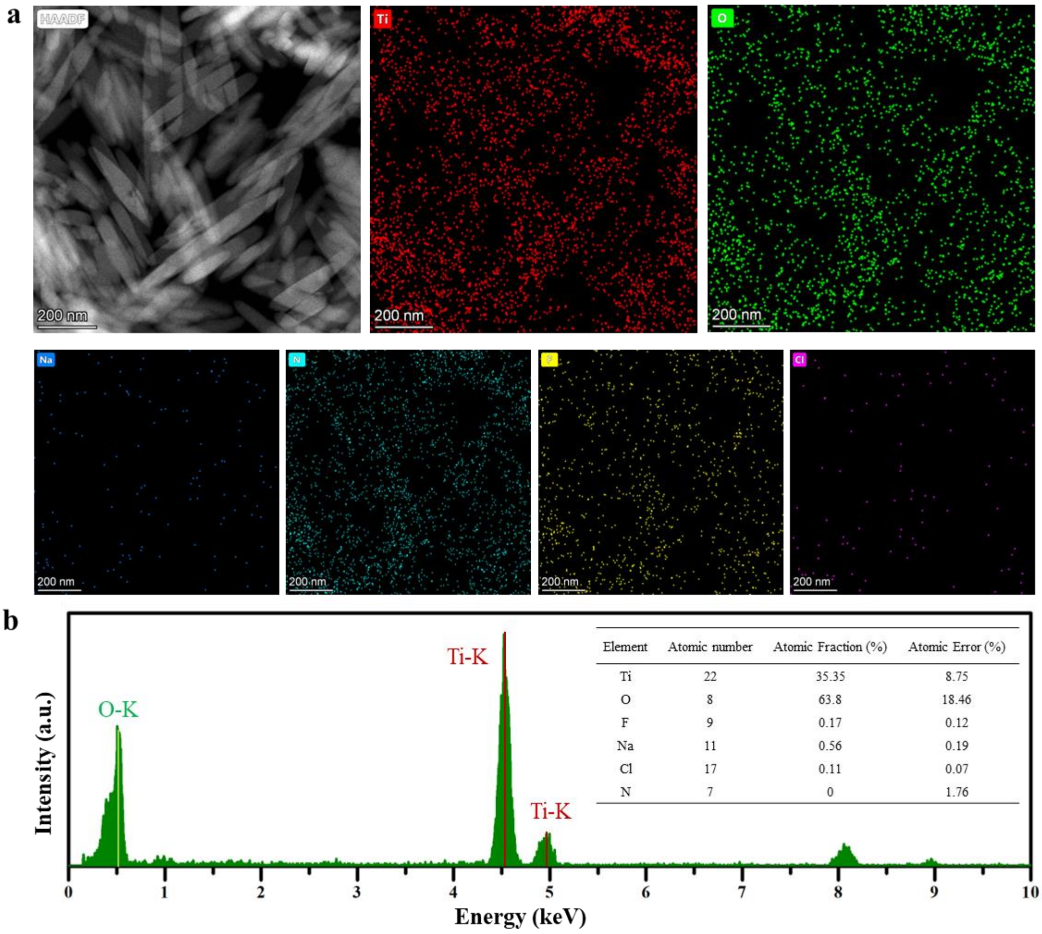

Figure R6. (Fig. 1 in SI) a. HAADF-STEM and EDS elemental mapping of Ti and O elements, and possible influencing elements; **b.** quantitative element diagrams of the prepared anatase nanorods from (a).

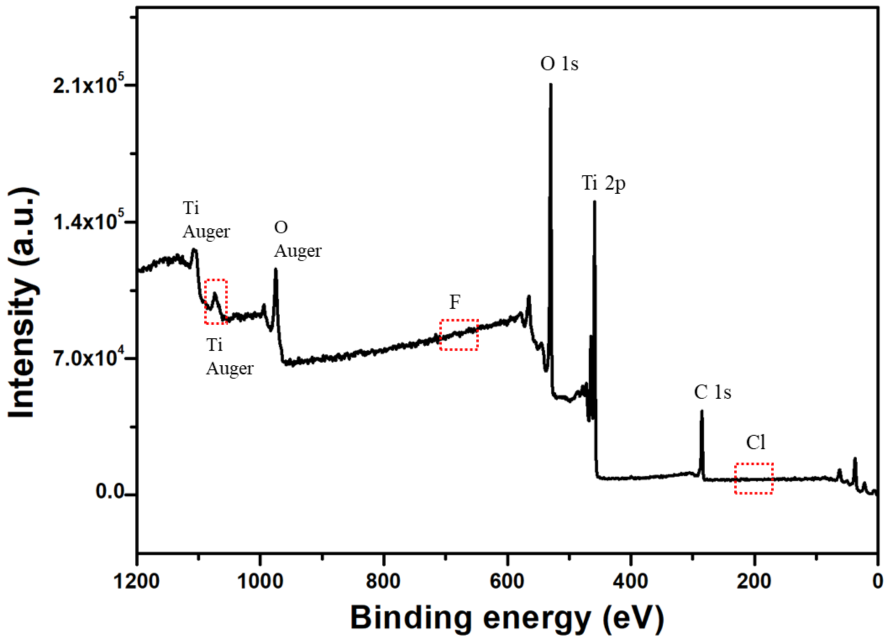

Figure R7. (Fig. 2 in SI) XPS spectra of the prepared anatase nanorods.

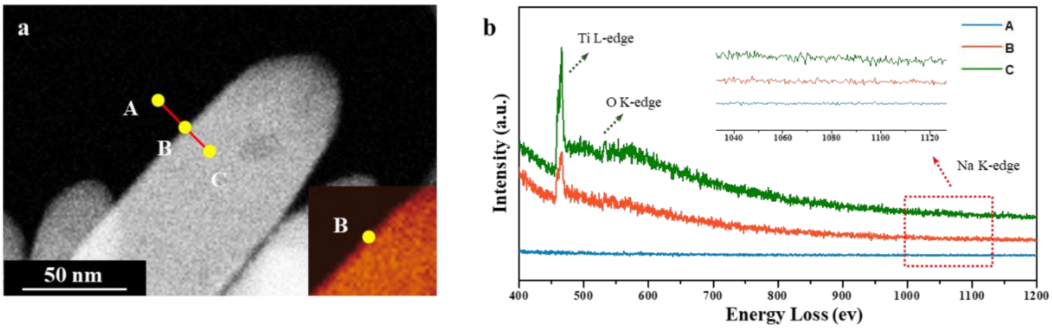

Figure R8. (Fig. 3 in SI) a. ADF STEM image showing the anatase TiO_2 nanorod configuration under vacuum at room temperature with EB irradiation. **b.** EELS spectra extracted from the yellow points in (a).

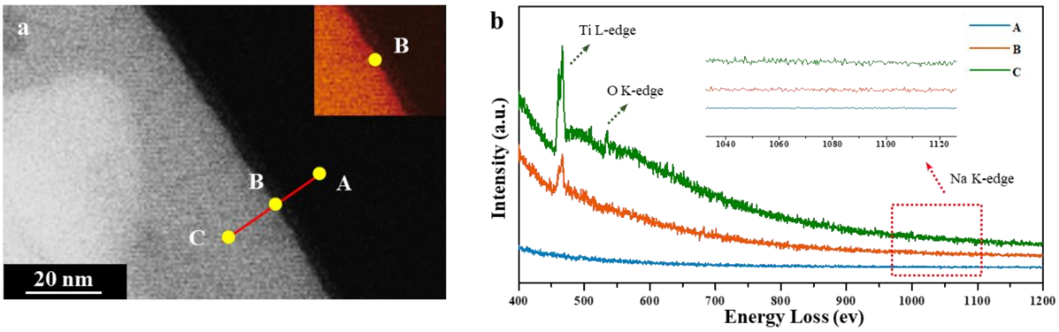

Figure R9. (Fig. 4 in SI) a. ADF STEM image showing the anatase TiO_2 nanorod configuration under vacuum at room temperature with EB irradiation. **b.** EELS spectra extracted from the yellow points in (a).

Comment #4: All the STEM observation of different surface restructuring and DFT calculations show are the relative energetics of low Miller-index surfaces of anatase – the surface energetics have been already well established. Are the authors claiming an "exceptional" stability due to the absence of (112) facets in the nanorods? There are neither analyses nor evidences presented in this manuscript that the absence/presence of such a controlling factor inhibits/promotes ART.

Response: We appreciate you for the insightful comments, which provide us opportunity to better clarify the scope and significance of our claims. Based on your comments, we have thoroughly revised the Introduction and Discussion of the manuscript. We agree that the relative surface energetics of individual low Miller-index anatase facets are well-established. However, we contend that the fundamental understanding of surface behavior under conditions that drive phase transformation remains incomplete: (1) The synergistic evolution and reconstruction mechanism between different crystal facets; (2) how these

surface evolution or reconstruction collectively affect the phase stability. We also sincerely apologize that our previous manuscript failed to clearly illustrate the mechanistic role of (112) facets and did not adequately link our experimental observations to the underlying phase transition mechanism. Actually, the "exceptional" stability we observe in isolated nanorods is therefore not only due to the absence of a single facet like (112), but also results from the kinetic inhibition of nucleation imposed by the restructured surfaces. In the revised manuscript, we have incorporated new discussions that explicitly connect the findings from our in situ experiments with the mechanism proposed in our conclusion.

As illustrated in Fig. R10a, the formation of (112) twin planes – widely recognized as preferential nucleation sites for the anatase-to-rutile transition (ART) – requires specific edge-to-edge connections between adjacent (101) facets. In our synthesized nanorods, which predominantly expose (100) facets, this particular stacking geometry is structurally suppressed (*Chem. Mater. 2018, 30, 1, 288–295*). This facet constraint directly links to the observed phase stability. Our in situ experiments confirm that individual nanorods resist ART up to 1250 °C, undergoing only surface reconstruction.

Conversely, as depicted in Fig. R10, rutile nucleation is only observed when multiple nanorods stack in the specific configuration needed to form (112)-like interfaces. As the number of stacked nanorods increases, the probability of 112 crystal plane formation increases, and thus the probability of phase transformation also increases. In our study, in-situ video observations clearly reveal the rapid migration of surface atoms. This phenomenon suggests that the surface mass transfer process is highly active at elevated temperatures, which, from a theoretical perspective, should facilitate diffusion and phase transitions.

Therefore, the "exceptional" stability stems not from general surface energetics, but from the kinetic suppression of ART nucleation due to the extensively restructured and stabilized surfaces, and the formation of the (112) crystal plane with a low occurrence probability – a controlling factor directly visualized and substantiated by our atomic-scale evidence. Our discovery provides atomic-scale insights into the critical influence of surface atomic structure and restructuring on the phase stability of nanomaterials. The proposed “surface locking” mechanism may serve as a fundamental principle for manipulating phase stability in the design of future high-temperature functional nanocrystalline materials.

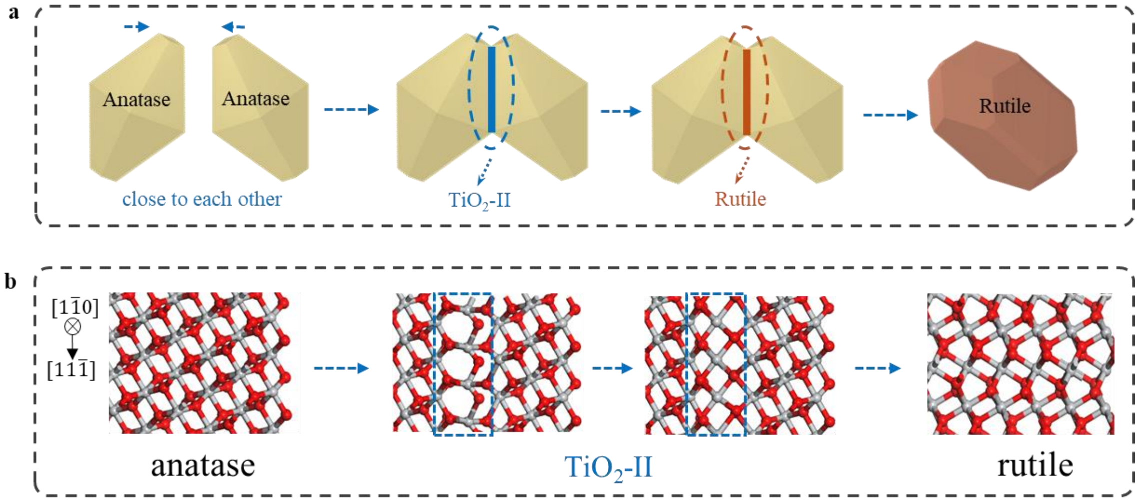

Figure R10. (Fig. 16 in SI) a. Schematic diagram of 112 crystal plane formation and rutile nucleation. **b.** Schematic diagram of phase transition from anatase to rutile. Red: O; Gray: Ti ; Blue dashed box: Ti and O after reconstruction [*J. Am. Chem. Soc. 2015, 137, 35, 11532* – *11539*].

Reviewer #2

General Comments: The authors report on high thermal stability of individual anatase TiO_2 nanorod single crystals with detailed characterisation using different electron microscopy methods. They observe a noteworthy stability at 1250 °C, and give convincing reasoning behind for the observed stability of the individual crystals. The data analysis and methodology are sound for the most part, with the exception of elemental analysis of the TiO_2 nanorods missing from the manuscript. Even though the results are convincing and relatively interesting, I do not believe this manuscript reports results that are significant enough to the related fields to merit publication in Nature Communications. I instead recommend publishing the results in a more specialised journal.

Response: Thank you very much for your time and thoughtful review of our manuscript. We sincerely appreciate your positive comments regarding our experimental results, particularly your acknowledgment that our results are "convincing and relatively interesting." We have carefully considered your concern regarding the absence of elemental analysis and conducted EDS, XPS and EELS analyses to confirm the purity of our samples, which further strengthen our findings. In addition, we would like emphasize the significance of our work:

Although the anatase-to-rutile phase transition (ART) has been investigated for many years, several fundamental aspects of its phase transition mechanism remain unclear. As you pointed out, “ART occurs over a wide temperature range, e.g., between approximately 600 °C and 1200 °C, depending on many factors,” which highlights the variability of experimental observations across different studies. Previous works have also investigated the influence of different factors on phase transitions, but mainly based on ex-situ characterization and theoretical calculations, lacking clear atomic-scale in-situ evidence. In our experiments, we integrated spherical aberration corrected environmental scanning transmission electron microscope, atomic-scale secondary electron surface imaging, and the construction of a highly regular model system, to clarify the critical role of the surface in ART process. Our in situ results provide the atomic insight for the surface structural evolution and high stability of individual anatase TiO_2 single crystal. This study also suggested that dynamic surface atomic restructuring not only reduces the total energy of the system but also kinetically inhibits surface rutile nucleation.

Comment #1: I elaborate on my reasoning here: The authors claim many times that they observe stability at temperatures exceeding typical anatase by 650 °C. However, this is a misleading claim since it only applies to pure bulk anatase. Anatase stability has been studied for more than 60 years in the literature, and the stability of the anatase phase is well known to depend greatly on impurities, the synthetic procedure, and the atmosphere during heat treatment. The reported values for anatase-to-rutile transformation indeed vary between 400–1200 °C in the literature (e.g. 10.1007/s10853-010-5113-0). The authors utilise a synthesis with Na-titanate precursors, and TiO2 with Na2O impurities have been long ago reported to show a phase transition temperature exceeding 900 °C. The fact that the individual nanocrystals in the manuscript show stability exceeding 1250 °C is not surprising, considering that anatase samples have been shown to remain stable at up to 1200 °C (10.1111/j.1151-2916.1965.tb14774.x).

Response: Thank you for your insightful comments and pointing out the lack of precision in our original statement regarding the phase transition temperature. We have thoroughly revised the relevant descriptions throughout the manuscript to make the expression more rigorous. We fully agree with your observation that reported transition temperatures vary widely—from 400 °C to 1200 °C, and that this variability was attributed to numerous factors including impurities, synthesis methods, and atmosphere, etc. We also acknowledge that the emphasis on the transition temperature of 1250 °C in our initial manuscript could be

misleading. In the revised manuscript, we have reframed the Abstract, Introduction and Discussion, to avoid overstating the specific temperature value. Instead, we focus the main discussion on the key scientific advances of this work: the distinct phase-transition behaviors observed between isolated and aggregated nanorods, and the surface atomic evolution process revealed by in situ observation.

Actually, the broad transition temperature range reported in the literature precisely underscores the fact that the fundamental factors controlling anatase stability are not yet fully understood. Although some general conclusions have been drawn, how these factors collectively govern the thermal stability of TiO_2 remains unclear. To bridge this gap, our study provides unprecedented in situ, atomic-level insight into this classic problem, through a state-of-the-art spherical aberration-corrected environmental scanning transmission electron microscope and high-resolution secondary electron surface imaging. By in situ investigating a well-designed model system—single-crystal nanorods with uniform dimensions, well-defined morphology, and high crystallinity—we have elucidated the intrinsic phase stability of individual nanorods at the atomic scale. Through a direct comparison between isolated nanorods and their overlapping aggregates, we have confirmed the key factor of inter-particle contact for nucleation. Our results reveal that the surface reconstruction could also exert a profound impact on phase stability, as it significantly enhances stability by effectively suppressing surface nucleation in individual nanoparticles.

As you mentioned, it is well documented that Na_2O impurities could significantly alter the phase transition temperature. In this work, although Na-titanate was used as the precursor, several measures were taken to exclude any consequential influence of sodium. The synthesized nanorods were first thoroughly washed with deionized water to remove any potential surface residues, which is a widely used procedure for removing surface contaminants (*Sci. Sin. Chim. 2014, 44,1618; Sci Rep. 2015, 5, 12143*). Subsequently, Energy-Dispersive X-ray Spectroscopy (EDS), X-ray Photoelectron Spectroscopy (XPS) and Electron Energy Loss Spectroscopy (EELS) analyses (Figures R6~R9) were conducted to determine the presence of residual sodium. In both EDS, XPS and EELS data, no obvious Na signals were observed (Figures R6~R9), indicating that they did not play a significant role in our study. which have been added in the Supplementary Information.

In the EDS analysis, no distinct signal corresponding to the Na element was observed within its characteristic energy range (1.04 – 1.06 keV), as the signal level remained consistent with the background. In the XPS spectrum, the peak position attributed to Na (1072 eV) is in close proximity to the Ti Auger electron peak

(1067 eV); based on binding energy comparison, this feature was assigned to the Auger peak of Ti rather than Na. To further investigate the presence of Na, EELS measurements were conducted at multiple locations across different nanorods. No discernible signal was detected at the expected energy position for Na (1072 eV). By integrating results from EDS, XPS, and EELS analyses, the potential interference from Na was systematically evaluated and effectively ruled out in this study.

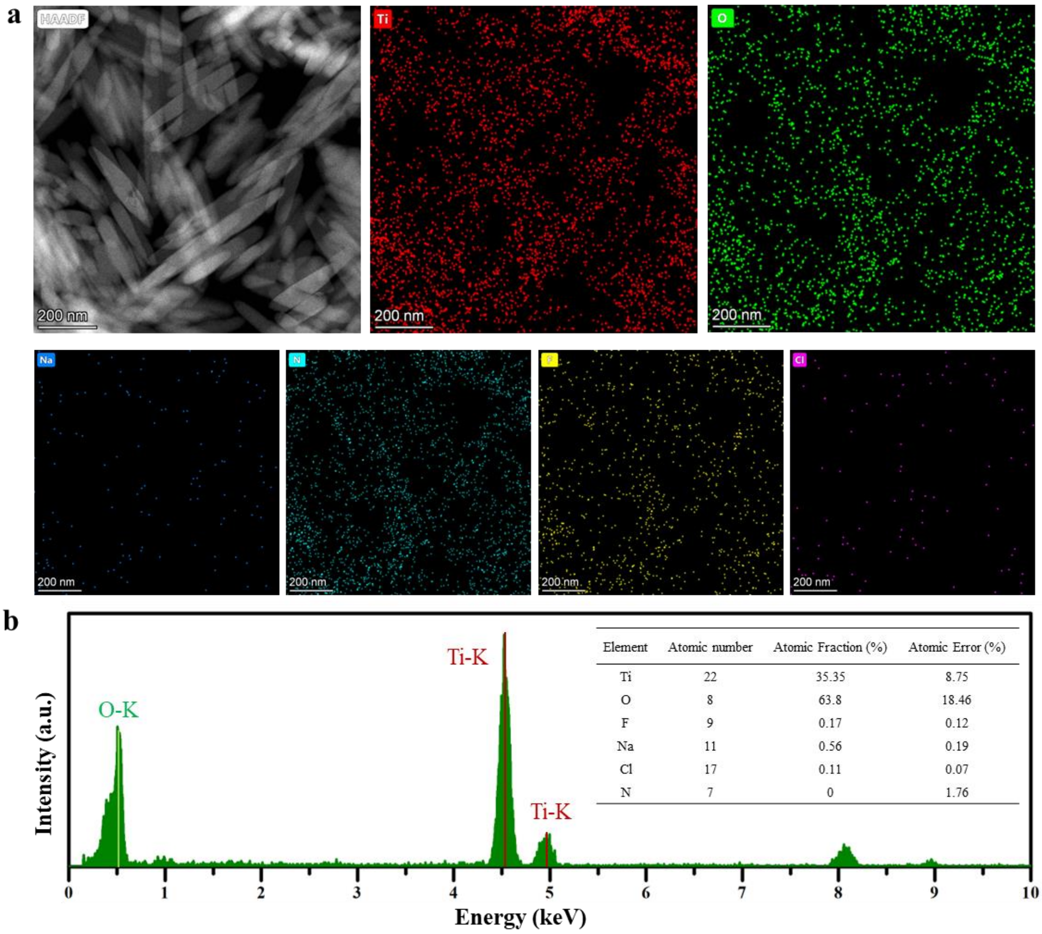

Element	Atomic number	Atomic Fraction (%)	Atomic Error (%)
Ti	22	35.35	8.75
O	8	63.8	18.46
F	9	0.17	0.12
Na	11	0.56	0.19
Cl	17	0.11	0.07
N	7	0	1.76

Figure R6. (Fig. 1 in SI) a. HAADF-STEM and EDS elemental mapping of Ti and O elements, and possible influencing elements; **b.** quantitative element diagrams of the prepared anatase nanorods from (a).

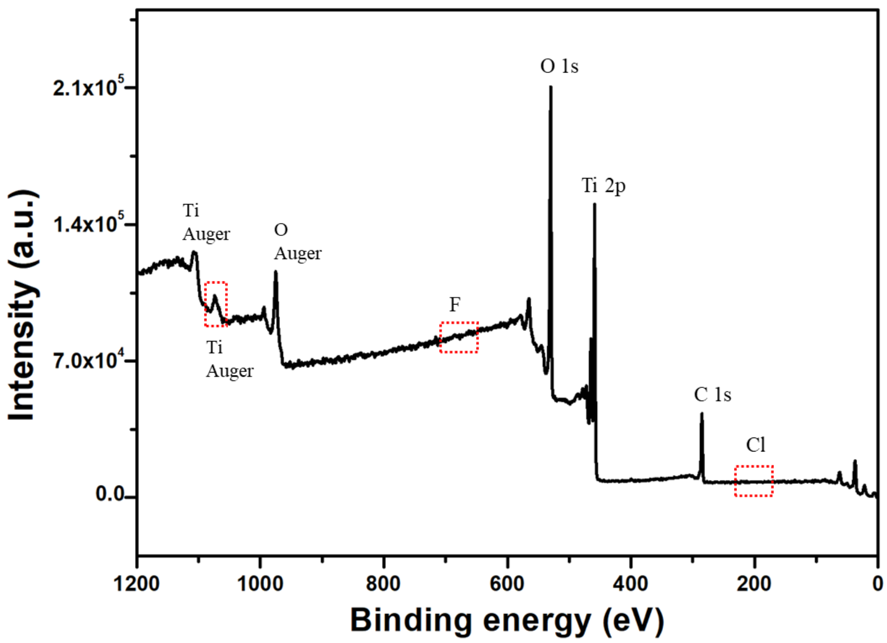

Figure R7. (Fig. 2 in SI) XPS spectra of the prepared anatase nanorods.

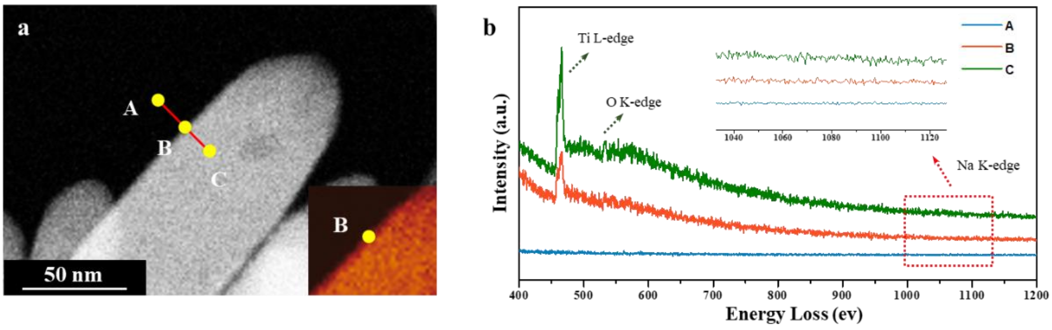

Figure R8. (Fig. 3 in SI) a. ADF STEM image showing the anatase TiO_2 nanorod configuration under vacuum at room temperature with EB irradiation. **b.** EELS spectra extracted from the yellow points in (a).

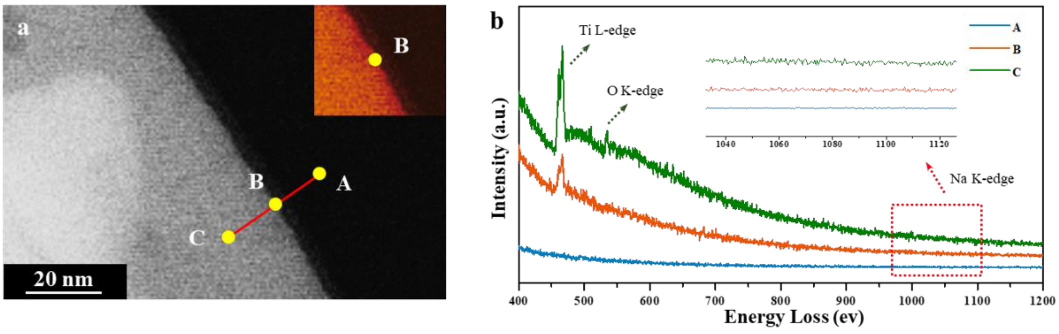

Figure R9. (Fig. 4 in SI) a. ADF STEM image showing the anatase TiO_2 nanorod configuration under vacuum at room temperature with EB irradiation. **b.** EELS spectra extracted from the yellow points in (a).

Comment #2: The fact that the annealing in the manuscript is performed in vacuo also affects the phase transformation, with reports spanning back 60 years how titanium interstitials retard the phase transformation due to a "structure stuffing effect" (10.1111/j.1151-2916.1965.tb14774.x). This is to my understanding the same process the authors call "surface locking".

Response: Thank you for your constructive comments and valuable suggestions. We agree with you that the vacuum conditions may also affect the phase transformation process. However, the specific role of vacuum annealing in phase transformation remains debated in the literature, and a clear consensus has yet to be established. For instance, several recent studies suggest that vacuum conditions may actually promote phase transformation by facilitating oxygen vacancy formation, rather than suppress it (*J. Alloy. Compd. 2025, 1039, 183313; Mater. Charact. 2025, 227, 115226*). It should be emphasized that all experiments in this study were conducted under identical vacuum conditions (pressure: 10^{-5} Pa). Nevertheless, our study is primarily focused on elucidating the key behavioral difference between isolated single-crystal nanorods and their aggregated counterparts. Our primary finding is that, under the same vacuum conditions, phase transformation occurs more readily in stacked nanorods.

Furthermore, the "surface locking" mechanism we proposed is distinct from the "structure stuffing effect" you mentioned. According to the reference you provided, the structure stuffing effect acts primarily as a kinetic factor by impeding atomic diffusion. In contrast, our results indicate that "surface locking" operates through a thermodynamic pathway by inhibiting surface nucleation processes. This interpretation is also supported by our in situ high-temperature observations, which reveal significant surface reconstruction—clear evidence that atomic diffusion remains rapid under our experimental conditions and is not kinetically limited (Figure 5, Videos 1~3). Therefore, our findings and proposed mechanism can be clearly distinguished from earlier explanations.

Comment #3: The reconstruction of TiO_2 nanocrystal to a majority of (101) surface instead of (112) surface is not surprising, and was published by many of the same authors 10 years ago (10.1021/acs.nanolett.5b03277). Furthermore, the importance of the (112) surface in the anatase-to-rutile transformation has been known for more than 20 years (10.1111/j.1151-2916.2001.tb00709.x). I agree with the authors assignment for reasons behind the retardation of the phase transformation, but do not think the

results are particularly significant to a wide audience.

Response: Thank you for the insightful comments and the references you provided. We are encouraged by your agreement with our assignment of the reasons behind the retardation of the phase transformation. We agree that our previous work (*10.1021/acs.nanolett.5b03277*) offers valuable insights into the high-temperature reconstruction of the anatase (001) surface. However, that study primarily focused on the (1×4) reconstruction of the (001) facet and did not address the behavior or dynamic role of the (101) surface under extreme conditions. We also agree with you that, the significance of the (112) surface in the anatase-to-rutile transition was proposed more than two decades ago. Nevertheless, the overall ART mechanism remains a subject of debate, as reflected in the diverse pathways reported in the literature. For instance, various studies have suggested phase transition routes involving brookite (Science, 1998) or TiO_2-II (Acta Mater., 2010), while work by Banfield, Liu, and others has strongly argued for the (112) surface as the exclusive nucleation site (*Am. Mineral.* 1999, 84, 871-876; *J. Am. Chem. Soc.* 2015, 137, 11532-11539). This persistent lack of consensus can largely be attributed to the absence of atomic-scale, in situ evidence capturing the structural evolution at high temperatures.

While ex situ studies and theoretical calculations have provided a foundational understanding of the phase transition mechanism, this work, by integrating advanced in situ and atomic-resolution secondary electron imaging with HAADF-STEM in a well-defined model system, offers direct dynamic evidence that significantly deepens the mechanistic understanding. Our observations provide atomic-scale visualization supporting the critical role of surface nucleation in phase transition. We believe this work contributes to clarifying long-standing academic debates in this filed, and deliver three key advances: First, by providing in situ atomic-level visualization, this work reveals the dynamic reconstruction and facet evolution mechanism of anatase TiO_2 at high temperatures, filling a critical gap in the understanding the structural evolution of anatase TiO_2. Second, building on this insight, we demonstrate how surface reconstruction can be utilized to inhibit phase transition nucleation, achieving exceptional phase stability in individual nanorods. Specifically, we discovered that the metastable surface configuration creates a protective "locking" effect, which fundamentally differs from previously reported mechanisms. These findings provide not only new fundamental insights into surface-mediated thermal stabilization but also a practical pathway for designing high-temperature nanomaterials through surface engineering. Third, although previous studies have reported phase transitions occurring over a relatively wide temperature range due to various influencing factors, this

work clearly demonstrates that surface reconstruction could play a critical role in enhancing stability by strongly suppressing surface nucleation.

Reviewer #3:

General Comments:

The experimental results of this manuscript are quite beautiful and instructive. Still, their presentation needs major revision: It is absolutely untrue that anatase is not stable in microscopic form -- only for nanomaterials, it converts to rutile irreversibly. Surface science work (ignored completely here) has clearly shown that antatase (101) single crystals are very stable, up to highest temperature. This is different from brookite, where the temperature-driven phase transition is indeed occuring at several hundred °C.

Response: Thank you for your positive comments on our experimental results and your constructive suggestions which significantly improve the quality of the manuscript. We sincerely apologize for the oversight and the inaccurate statement in our original manuscript. We fully agree with you that anatase (101) single crystals are very stable in microscopic form, and our results provide the atomic insight for its high stability. According to your advices, the corresponding statements in the manuscript has been thoroughly revised by incorporating with new references and discussions.

Comment #1: What the authors observe - in a very nice and detailed fashion is just the restructuring of high-energy surfaces to the lowest-energy one, the 101 surface. It has been known since more than 25 years (see DFT calculations of the Wulff constrution by Selloni, for example), that this surface is exceedingly stable. It is no wonder, then, - but still nice to see - that the relatively big 'nanograins' of anatase show faceting of their high-energy surfaces to 101 facets. Also, a well known and entirely expected phenomenon based on decades of surface science research.

Response: We appreciate your positive comments on our results, and also thank you for the accurate re-framing of our observations. We have made corresponding revisions based on your comments. Specifically, we have incorporated a discussion on the Wulff construction for anatase TiO_2 and have cited the pioneering theoretical work of Selloni and colleagues (e.g., A. Selloni et al., *Phys. Rev. Lett., 1998, 81, 2954*) and other key experimental surface science studies that established the exceptional stability of the anatase (101) surface.

Comment #2: Because many statements are ignoring prior knowledge on this system they are misleading. The good news is that, in my opinion, the experiments do not need to be repeated or extended -- just re-interpreted in the proper way. Without these major revisions, I cannot recommend publication of this paper.

Response: We sincerely thank the reviewer for this critical insight. We acknowledge and regret that our original interpretation overlooked significant prior studies, which led to misleading statements. We are greatly encouraged by the comment that the experimental data themselves are sound. Following your advice, we have undertaken a major reinterpretation of our results. The revised manuscript now situates our findings within the proper conceptual framework in line with your guidance, with carefully consideration of the established surface science research. We have meticulously gone through the manuscript—particularly the Introduction and Discussion sections—to ensure all statements are now aligned with the established understanding. We believe these revisions have fully addressed your concerns and substantially strengthened this work.

Point-by-point response to Reviewers

Reviewer #1:

General comments: The manuscript titled, "Excellent Phase Stability of Individual Anatase TiO_2 Nanocrystals via Reshaping and Surface Restructuring," by X. Guo et al. has been reviewed.

This revised manuscript focuses on the mechanism of phase stability in individual anatase TiO_2 nanorods. Using in situ atomic-resolution STEM, the authors show that high-temperature stability arises from surface restructuring, where facets evolve into low-energy (101) surfaces, creating a "surface-locking" effect that kinetically inhibits rutile nucleation. In contrast, aggregated nanorods transform readily, highlighting the role of surface dynamics in phase stability.

However, the central observation and interpretation largely confirm what is already well established. It is known that among low-index anatase surfaces, only the (112) facet provides nucleation sites for the anatase-to-rutile transition (Zhu et al., J. Am. Chem. Soc. 137, 11532 (2015)). Restructuring of (101) and (100) surfaces costs ~0.71 eV and ~0.82 eV, respectively, compared to ~0.45 eV for (112), and only (112) surfaces allow restructuring to propagate into the bulk. Therefore, in the absence of (112) facets, isolated nanorods are expected to remain anatase. The STEM observations and DFT calculations presented here essentially validate this known model rather than providing fundamentally new insights.

In summary, while the experiments are carefully executed, the mechanistic conclusions do not advance the current understanding sufficiently for publication in Nature Communications. The work would be more appropriate for a specialized materials science journal.

Response: We sincerely thank you for your time and thoughtful evaluation of our revised manuscript, and also appreciate your accurate summary of our core findings and your acknowledgment of the carefully executed experiments. We also appreciate you highlighting the foundational study by Zhu et al. (*J. Am. Chem. Soc.* **2015**), which established the critical thermodynamic role of the (112) facet in the anatase-to-rutile transformation (ART). We acknowledge that in our previous draft, the presentation was not sufficiently clear in distinguishing our new findings from established thermodynamic models. We respectfully wish to clarify that our work does not merely validate Zhu's static thermodynamic model, but rather reveals a previously unrecognized dynamic kinetic mechanism of the "surface-locking" effect. In addition, it should be emphasized that in practice, many complex thermodynamic and kinetic factors affect the phase thermostabilities, the dominant role in the practice experiments remains elusive (details are discussed below).

To bridge this gap, our study provides unprecedented in situ, atomic-level insights of both surface and bulk structural evolution of TiO_2 nanocrystal, through the state-of-the-art spherical aberration-corrected environmental STEM and advanced high-resolution secondary electron surface imaging. By investigating a well-designed model system — single-crystal nanorods with uniform dimensions, well-defined

morphology with multiple facets exposed, and high crystallinity — we elucidate the atomic structural evolution and intrinsic phase stability of individual anatase TiO_2 nanorods. Direct comparisons between isolated nanorods and their aggregates confirm the critical role of interparticle contact in nucleation, while surface reconstruction further enhances stability by suppressing surface nucleation in isolated nanoparticles. Our work provides direct atomic-level evidence for the structural evolution of anatase TiO_2, and clarifies long- standing debates on phase stability in nanocrystalline TiO_2 systems. We have significantly revised the manuscript to clarify these crucial distinctions.

Clarifying the Dominant Role in Nanosized Anatase TiO_2 System: Phase transitions in TiO_2 nanocrystal system are governed by a complex interplay of thermodynamic and kinetic factors. While the (112) twin boundary is a widely accepted nucleation site from a thermodynamic perspective based on theoretical calculations, competing models have also been proposed [e.g., direct surface nucleation pathways, defect/oxygen vacancy driven model and disordered interface model (*J. Mater. Res.*, **2000**, 15, 437-448.; *Phys. Chem. Chem. Phys.*, **2023**, 25, 3199-3210; *J. Eur. Ceram. Soc.*, **2006**, 26, 1527-1534; *J. Mater. Sci.*, **2011**, 46, 855-874; *Mater. Sci.*, **2010**, 46, 1780–1788)]. Furthermore, numerous kinetic factors, such as atomic diffusion rate, defect Concentration, etc., (Hanaor & Sorrell., J. Mater. Sci., 2011, 46, 855-874; Shannon & Pask., J. Am. Ceram. Soc, 1965, 48, 391-398; Gribb & Banfield., Am. Mineral., 1997, 82, 717-728; Reidy, Holmes & Morris., J. Eur. Ceram. Soc., 2006, 26, 1527-1534), are often proposed as the rate-limiting steps controlling the transition. The multiplicity of potential influences underscores that a definitive mechanistic understanding cannot be derived solely from theoretical models or ex situ studies. To bridge this fundamental gap, our work provides direct, atomic-scale real time experimental validation under elevated temperature condition, thereby elucidating the actual dominant mechanisms at play.

Direct In Situ Atomic Level Experimental Evidence: Our study presents the first direct, in situ atomic-resolution observations—utilizing HAADF-STEM, BF-STEM, and secondary electron imaging—of individual, faceted anatase TiO_2 nanorods evolving at high temperatures. This multi-dimensional approach allows us to comprehensively decouple and clarify both surface restructuring dynamics and internal structural integrity. These combined atomic-level insights uniquely elucidate the dynamic evolution of different surfaces and the bulk phase under elevated temperatures, reveal the mass transport mechanisms during transformation, and fully delineate the decisive impact of surface status and its reconstruction on the intrinsic thermal stability of individual nanocrystals.

Critical Role of Isolation vs. Aggregation: We directly demonstrate that isolated nanorods exhibit dramatically enhanced phase stability compared to their aggregated counterparts. This critical finding highlights the overarching role of unhindered surface dynamics in phase stability—a dimension not directly addressed in prior literature, which has primarily focused on the intrinsic properties of isolated facets. Although interfaces are thermodynamically favorable for nucleation, other defects or surface sites are also frequently theorized as potentially active. Our experiments provide definitive, visual evidence that in our system, the inter-particle interface is the dominant nucleation site, overriding other potential

candidates. This key mechanistic distinction, central to predicting real-world material behavior, is extraordinarily difficult to ascertain through computation alone.

Broader Implications: Beyond elucidating the restructuring mechanisms and intrinsic thermostability of TiO2 nanocrystals, this work also establishes an effective pathway for simultaneously probing the complex structural evolution of nanomaterials across both surface and bulk regions. In addition, previous theoretical work proposed possible mechanisms for the phase transition. Our discovery does not merely validate an established mechanism with atomic-scale, dynamic evidence; it provides a comprehensive experimental assessment of the thermal stability in both isolated and aggregated nanostructures, conclusively establishes the critical role of surface restructuring. By demonstrating that surface reconstruction can kinetically stabilize a metastable phase, we suggest a strategy may potentially extend to other nanomaterial systems.

Reviewer #2

General Comments: The authors report on high thermal stability of individual anatase TiO_2 nanorod single crystals with detailed characterisation using different electron microscopy methods. The authors have revised their manuscript by modifying the discussion (albeit only slightly) and performing elemental analysis of their nanoparticles. The elemental analysis gives required information on the composition of the nanoparticles. Even with the added analysis and corrections to the text, previously incorrect statements remain, such as "The phase structure of anatase TiO_2 could transformed into rutile around ~600 °C", which is not an apples-to-apples comparison to the individual nanoparticles here. They have for the most part added the references I required, but the amount of discussion on the importance of their results relative to the existing literature remains lacking. Even though the measurements are quite interesting, I do not see a reason to change my previous assessment that the results in this manuscript are not significant enough to the related fields to merit publication in Nature Communications. I instead recommend publishing the results in a more specialised journal.

Response: We sincerely thank you for your thorough and constructive evaluation of our revised manuscript. We appreciate your recognition that our experimental measurements are quite interesting, and we are grateful for your valuable feedback. We appreciate your acknowledgment of the interesting nature of our measurements, the high thermal stability demonstrated in our individual single crystals, and the value of our added elemental analysis. We have carefully considered your remaining concerns and have made further significant revisions to ensure the importance of our findings is properly contextualized within the existing literatures. We apologize for the imprecise statement concerning the phase transition temperature in our previous version. You are correct that the literature value predominantly applies to aggregated nanoparticle powders or bulk materials, and using it as a direct baseline for the isolated single-crystal nanorods studied here is not an "apples-to-apples" comparison. In fact, this profound discrepancy in transition temperatures between isolated and aggregated systems is exactly the central phenomenon our study elucidates.

We respectfully emphasize that, in practice, it is precisely because so many complex thermodynamic and kinetic factors affect phase thermostability that the dominant mechanism in experimental settings remains elusive. This complexity is reflected in the extremely broad range of transition temperatures reported in the literatures. The uncertainty of these diverse factors' influence stems from the lack of direct, atomic-level experimental evidence—a critical gap that precisely underscores the fundamental significance of our study.

Our work provides the first in situ, atomic-scale visualization of the structural transition in well-defined, isolated nanorods, offering direct evidence that resolves long-standing debates regarding the intrinsic stability and reconstruction dynamics of anatase TiO_2. By investigating a well-defined model system—isolated single-crystal nanorods with uniform dimensions—we effectively established an "ideal" baseline to observe the intrinsic thermal stability of anatase TiO_2 without the interference of interparticle interfaces. By systematically comparing isolated versus aggregated nanostructures, we directly address the role of interparticle contact, a factor often invoked but seldom demonstrated with such spatial and temporal resolution. More importantly, we establish surface reconstruction as a generalizable kinetic stabilization mechanism, advancing the design principles for thermally stable nanocrystalline materials. Our work bridges the gap between the behavior of an "isolated apple" (intrinsic stability via surface reshaping) and a "basket of oranges" (aggregation-driven transformation). This clear, atomic-level decoupling provides fundamental insights into predicting and controlling real-world material behaviors under thermal stress. We have significantly revised the manuscript to explicitly address the comparison standards and clarify this core mechanism. We believe these unique insights elevate the contribution of our study beyond incremental characterization and offer a foundational advance worthy of publication in *Nature Communications*. We have revised the manuscript to clarify these distinctions and explicitly address the comparison standards. The key advances are summarized below:

Phase transitions in TiO_2 nanocrystal system are governed by a complex interplay of thermodynamic and kinetic factors. While the (112) twin boundary is a widely accepted nucleation site from a thermodynamic perspective based on theoretical calculations, competing models have also been proposed (e.g., direct surface nucleation pathways, defect/oxygen vacancy driven model and disordered interface model [Zhang & Banfield., J. Mater. Res., 2000, 15, 437-448., Lu. et al., Phys. Chem. Chem. Phys., 2023, 25, 3199-3210; Reidy, Holmes & Morris., J. Eur. Ceram. Soc., 2006, 26, 1527-1534; Hanaor & Sorrell., J. Mater. Sci., 2011, 46, 855-874; Lee & Zuo., Mater. Sci., 2010, 46, 1780–1788]). Furthermore, numerous kinetic factors, such as atomic diffusion rate, defect Concentration, etc., (Hanaor & Sorrell., J. Mater. Sci., 2011, 46, 855-874; Shannon & Pask., J. Am. Ceram. Soc, 1965, 48, 391-398; Gribb & Banfield., Am. Mineral., 1997, 82, 717-728; Reidy, Holmes & Morris., J. Eur. Ceram. Soc., 2006, 26, 1527-1534), are often proposed as the rate-limiting steps controlling the transition. The multiplicity of potential influences underscores that a definitive mechanistic understanding cannot be derived solely from theoretical models or ex situ studies. To bridge this fundamental gap, our work provides direct, atomic-scale real time experimental validation under elevated temperature condition, thereby elucidating the actual dominant mechanisms at play.

Direct In Situ Atomic Level Experimental Evidence: Our study presents the first direct, in situ atomic-resolution observations—utilizing HAADF-STEM, BF-STEM, and secondary electron imaging—of individual, faceted anatase TiO_2 nanorods evolving at high temperatures. This multi-dimensional approach allows us to comprehensively decouple and clarify both surface restructuring dynamics and internal structural integrity. These combined atomic-level insights uniquely elucidate the dynamic evolution of different surfaces and the bulk phase under elevated temperatures, reveal the mass transport mechanisms during transformation, and fully delineate the decisive impact of surface status and its reconstruction on the intrinsic thermal stability of individual nanocrystals. This work also establishes an effective pathway for simultaneously probing the complex structural evolution of nanomaterials across both surface and bulk regions.

Critical Role of Isolation vs. Aggregation: We directly demonstrate that isolated nanorods exhibit dramatically enhanced phase stability compared to their aggregated counterparts. This critical finding highlights the overarching role of unhindered surface dynamics in phase stability—a dimension not directly addressed in prior literature, which has primarily focused on the intrinsic properties of isolated facets. Although interfaces are thermodynamically favorable for nucleation, other defects or surface sites are also frequently theorized as potentially active. Our experiments provide definitive, visual evidence that in our system, the inter-particle interface is the dominant nucleation site, overriding other potential candidates. This key mechanistic distinction, central to predicting real-world material behavior, is extraordinarily difficult to ascertain through computation alone.

Reviewer #3:

General Comments:

The manuscript has been improved; it no longer overlooks long-established concepts regarding the surface energy of nanoparticles in the anatase phase. It is a nice confirmation of these concepts and indeed interesting to specialists. It is not of appropriate generality and impact for a publication in Nature Communications, however.

Response: We sincerely thank you for your constructive feedback and for acknowledging the improvements in our revised manuscript. We appreciate the recognition of our work to be a "nice confirmation" of established surface energy concepts and interesting to the field. We also carefully noted your concerns regarding the generality and broad impact of our findings for publication in Nature Communications. However, we respectfully wish to clarify the significance of our work, which were not well illustrated in our previous version. It should be emphasized that, our work is not simply to confirm the existing theory, but also clarify the controversial concerning the thermostability in anatase TiO_2 nanocrystal. In practice, it is precisely because so many complex thermodynamic and kinetic factors affect phase thermostability that the dominant mechanism in experimental settings remains elusive. This complexity is reflected in the extremely broad range of transition temperatures reported in the literature. The uncertainty of these diverse factors' influence stems from the lack of direct, atomic-level experimental

evidence—a critical gap that precisely underscores the fundamental significance of our study. Our work provides the first in situ, atomic-scale visualization of the structural transition in well-defined, isolated nanorods, offering direct evidence that resolves long-standing debates regarding the intrinsic stability and reconstruction dynamics of anatase TiO_2. By systematically comparing isolated versus aggregated nanostructures, we directly address the role of interparticle contact, a factor often invoked but seldom demonstrated with such spatial and temporal resolution. More importantly, we establish surface reconstruction as a generalizable kinetic stabilization mechanism, advancing the design principles for thermally stable nanocrystalline materials. We believe these unique insights elevate the contribution of our study beyond incremental characterization and offer a foundational advance worthy of publication in *Nature Communications*. We have revised the manuscript to clarify these distinctions and explicitly address the comparison standards.
